# Deletion Analysis of Phase Separation, Amyloid Formation and Prion Propagation by the Intrinsically Disordered Region of Yeast Sup35 Protein

**DOI:** 10.3390/ijms27177516

**Published:** 2026-08-22

**Authors:** Anastasia V. Grizel, Natalia A. Gorsheneva, Ismat Jahan Anee, Kristupas Paulius, Konstantin Y. Kulichikhin, Aleksandr A. Rubel, Yury O. Chernoff

**Affiliations:** 1 School of Biological Sciences, Georgia Institute of Technology, Krone EBB, 950 Atlantic Dr. NW, Atlanta, GA 30332-2000, USA; anastasiia.grizel@biosci.gatech.edu (A.V.G.); ianee3@gatech.edu (I.J.A.); kristupas.paulius@gatech.edu (K.P.); 2 Laboratory of Amyloid Biology, St. Petersburg State University, 7/9 Universitetskaya emb, 199034 St. Petersburg, Russia; natalia.gorsheneva@mail.ru (N.A.G.); konstantin_kulichikhin@yahoo.com (K.Y.K.); a.rubel@spbu.ru (A.A.R.)

**Keywords:** IDRs, phase separation, amyloid, prion, [*PSI*^+^], Sup35, *Saccharomyces cerevisiae*, osmotic stress

## Abstract

Protein intrinsically disordered regions (IDRs) play important biological roles despite lacking stable structures. IDRs drive the formation of both biomolecular condensates via liquid–liquid phase separation (LLPS) and solid fibrous amyloid aggregates. Amyloids can be pathogenic and may exhibit self-perpetuating (prion) properties. Relationships between LLPS and the amyloid-forming and prion-propagating abilities of IDRs remain poorly understood. The N-proximal IDR of the yeast translation termination factor eRF3 (Sup35) can form both liquid condensates and heritable amyloid-based prions and serves as a powerful model for investigating these phenomena due to the availability of simple phenotypic, cytological and biochemical assays. Deletion analysis demonstrates that the N-proximal prion domain (Sup35N) of Sup35 is sufficient for chaperone-dependent prion propagation and that various regions of this domain show differential impacts on LLPS, amyloid aggregation, and prion inheritance. Specifically, the N-terminal NQ-rich stretch and the region of oligopeptide repeats are the most important contributors to the LLPS and formation of amyloid fibrils, while oligopeptide repeats and the C-terminal region of Sup35N are crucial for prion inheritance. Contrary to previous reports, the NQ-rich stretch is not required for prion formation and inheritance in yeast. Our data indicate that, in addition to amino acid composition, specific sequence motifs control reversible and heritable assemblies of Sup35.

## 1. Introduction

Intrinsically disordered regions (IDRs) of proteins lack a stable three-dimensional structure and are often enriched in low-complexity sequences. The importance of IDRs is signified by evidence of them performing important cellular roles, thus expanding the functional versatility of proteins [1]. Many functions of IDRs rely on phase separation, a process in which proteins and other biomolecules separate from the surrounding cellular milieu to form liquid- or gel-like assemblies known as condensates, or membraneless organelles [2,3]. By concentrating functionally related molecules, phase separation can promote or regulate biochemical reactions [4]. In some cases, IDRs can also promote amyloid formation, a process in which proteins assemble into fibrillar polymers held together by intermolecular β-sheets [5]. Such aggregates are associated with a variety of human diseases, including Alzheimer’s disease (Aβ peptide and MAPT, or tau protein), Parkinson’s disease (α-synuclein), amyotrophic lateral sclerosis (TDP-43 and FUS), and type 2 diabetes (islet amyloid polypeptide, IAPP). Notably, all these proteins can also undergo LLPS [5]. However, relationships between LLPS and amyloid formation remain poorly understood.

A growing body of evidence suggests that in some cases, LLPS precedes amyloid aggregation and could therefore be viewed as an intermediate stage in the amyloid generation pathway, so that increased local concentrations of IDR-containing proteins may facilitate their conversion to amyloid fibrils [6,7,8,9,10]. However, LLPS is widespread in living cells and involves a variety of proteins, whereas amyloid aggregation remains a comparatively rare event. This disparity argues against the notion that biomolecular condensates typically serve as amyloid intermediates. At present, direct evidence supporting condensate-to-amyloid conversion in vivo remains limited. Moreover, several in vitro studies indicate that condensates can interfere with amyloid formation. For example, sequestration of Aβ42 or TIA-1 into condensates inhibits fibril formation [11,12]). Indeed, condensates may prevent amyloid aggregation by favoring intermolecular interactions that interfere with amyloid assembly. In this scenario, pathological amyloid aggregation may arise when a balance between liquid–liquid and liquid–solid transitions is perturbed by physiological or environmental factors, mutations, aberrant phase behavior, and/or alterations in condensate composition. This raises the question of how the balance between functional protein condensation and often (although not necessarily) pathogenic amyloid formation is modulated in vivo and whether the same protein regions contribute to both processes.

**Figure 1:**
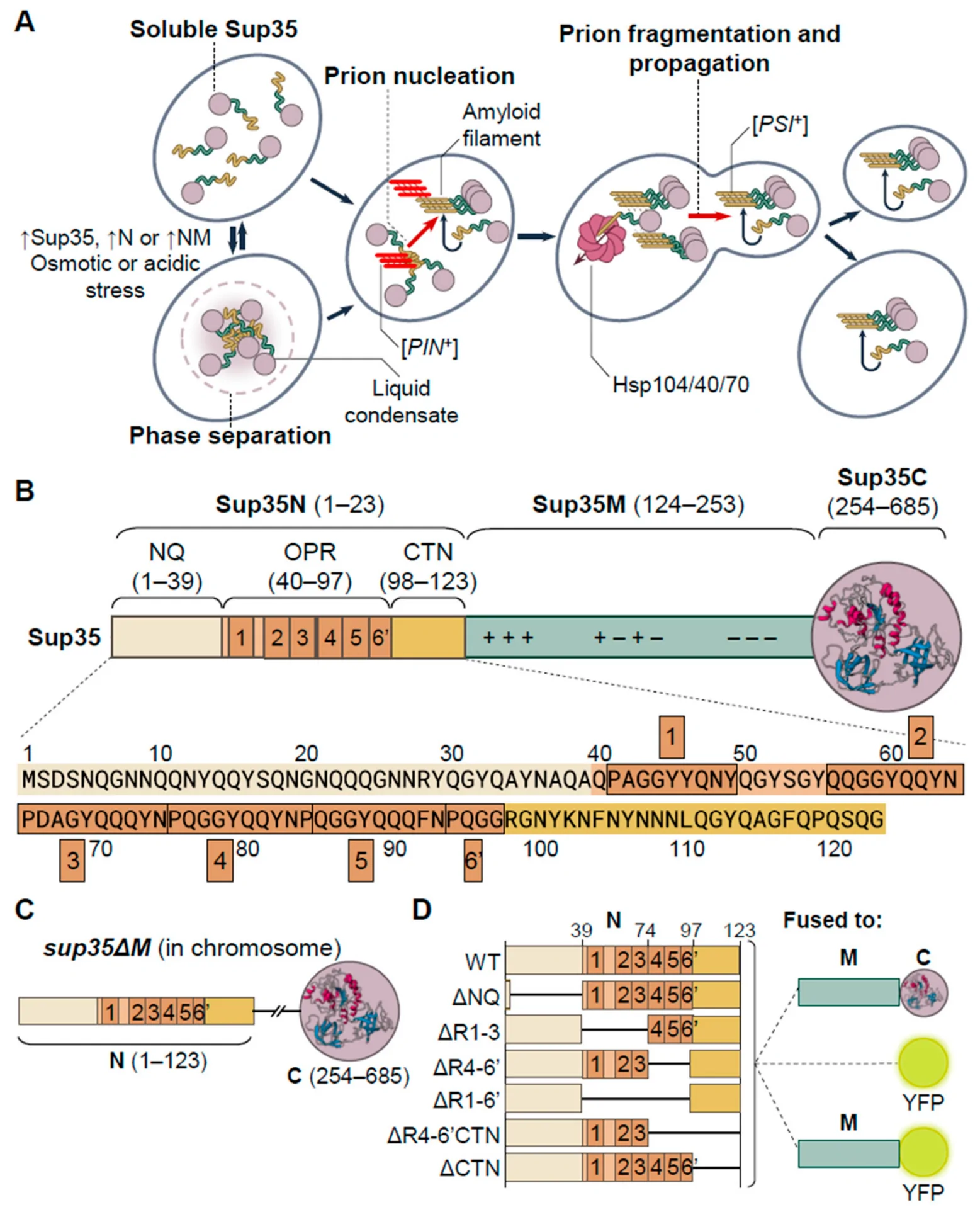
Aggregation and structural organization of *Saccharomyces cerevisiae* Sup35 protein. (**A**) Formation and propagation of liquid and solid assemblies by Sup35 protein. Here and further, protein overproduction is indicated by an upward arrow. (**B**) Domain organization of the Sup35 protein. Regions of Sup35N are designated as NQ (NQ-rich stretch), OPR (oligopeptide repeat region), and CTN (C-terminal region of N). Positively and negatively charged residues are indicated by “+” and “−”, respectively. The aa sequence of Sup35N is shown. Brown rectangles indicate imperfect oligopeptide repeats. Numbers located over and under drawings correspond to aa positions; numbers in boxes designate individual repeats, with incomplete sixth repeat designated as 6′. (**C**) Structure of the Sup35ΔM deletion variant encoded by a chromosomal allele. (**D**) Deletion derivatives of Sup35N, fused to either YFP, M-YFP or MC.

*Saccharomyces cerevisiae* Sup35 protein, a counterpart of eukaryotic release factor eRF3, that acts together with Sup45 (eRF1) in translation termination [13], has recently emerged as a convenient model for studying the in vivo roles of IDRs in both phase separation and amyloid aggregation. Sup35 can exist in several states in yeast cells (Figure 1A). Under normal conditions, it is a soluble cytosolic protein; however, in response to stress, such as cytosol acidification and severe energy depletion or hyperosmotic stress, it can undergo LLPS and form liquid condensates [14,15,16]. In the presence of a pre-existing heterotypic amyloid, most commonly a prion form of Rnq1 protein termed [*PIN*^+^], Sup35 can convert into an amyloid; this process is greatly facilitated by the transient overproduction of Sup35 or its prion domain (PrD) [17,18]. In vivo, Sup35 amyloid aggregates are fragmented by the Hsp104/Hsp70/Hsp40 chaperone machinery, generating seeds that immobilize a newly synthesized Sup35 protein and thus initiate growth of new amyloid fibrils. This results in the propagation and proliferation of a Sup35 amyloid state and its inheritance in cell divisions, thus establishing a heritable protein-based non-Mendelian element, designated as yeast prion [*PSI*^+^] [19]. Conversion of Sup35 into a prion state reduces its access to ribosomes, thereby causing nonsense suppression due to translational readthrough of stop codons [17]. This phenomenon provides the basis for a robust phenotypic assay of [*PSI*^+^] in yeast, based on partial recovery of the synthesis of a functional protein from ORF that is interrupted by a premature stop codon, such as *ade1-14* (UGA). *Ade1-14* cells lacking the Sup35 prion ([*psi*^−^]) do not grow on medium lacking adenine (-Ade) and accumulate red pigment derived from an intermediate of the adenine biosynthetic pathway on rich (YPD) medium, whereas [*PSI*^+^] cells exhibit growth on -Ade (Ade^+^ phenotype) and form white or light-pink colonies on YPD [19]. This assay also allows for discrimination between amyloid polymorphs with different fragmentation and propagation efficiencies, termed prion variants” or “strains” [20]. Stronger [*PSI*^+^] variants are fragmented more efficiently, producing seeds at higher numbers, which results in greater depletion of the pool of functional Sup35 and higher readthrough, thus causing better growth on -Ade, lighter color on YPD, and more efficient transmission in cell divisions, as compared to weaker [*PSI*^+^] variants. These features make the Sup35/[*PSI*^+^] a tractable and informative model for dissecting the mechanisms of amyloid formation and propagation.

The Sup35 protein is composed of the essential C-terminal region (Sup35C), encompassing amino acids (aas) 254–685, having a well-defined stable folded structure and responsible for the translation termination function, and a long IDR (aas 1–253) including two domains: the N-proximal PrD (Sup35N, aas 1–123), responsible for prion formation and propagation and for LLPS in response to osmotic stress, and the middle domain (Sup35M, aas 124–253) that contains clusters of positively and negatively charged residues, is thought to promote solubility, and acts as a pH sensor (Figure 1B) [14,15,16,21]. Sup35N shows low-complexity features: it is highly enriched in Q,N, aromatic (mostly Y), and G residues, contains very low proportion of charged residues, and can be further subdivided into an NQ-rich region (aas 1–39), a region containing 5.5 imperfect oligopeptides (OPR; aas 40–97), and a C-terminal segment (CTN; aas 98–123) (Figure 1B). Previous studies implied that the NQ-rich region is critical for amyloid and prion formation [22,23,24], whereas the OPR and CTN influence chaperone-dependent fragmentation and prion propagation [24,25,26,27]. However, “scrambled” Sup35 derivatives with the same aa composition but reshuffled sequences have been found to be typically able to form and propagate a prion state in yeast [28,29], thus questioning the specific roles of Sup35N subregions in these processes. To our knowledge, the impact of specific Sup35N subregions on LLPS has not been addressed.

Here, we applied a systematic deletion analysis of the Sup35 IDR (including Sup35 PrD) to compare the roles of its subregions in LLPS, amyloid assembly, prion nucleation, and prion propagation in vivo for one and the same set of constructs and conditions. We found that these processes are controlled by partially overlapping or redundant but functionally distinctive subregions, enabling us to distinguish between general and process-specific sequence requirements and shed light on underlying molecular mechanisms.

## 2. Results

### 2.1. Genetic Constructions Employed in This Work

In order to determine the contribution of different regions of the Sup35 IDR to LLPS, amyloid formation, the ability to nucleate a prion, and prion propagation, we constructed a series of deletions within the IDR-coding region. These included the *SUP35ΔM* allele lacking the Sup35M-coding region (Figure 1C) and *SUP35* alleles lacking: the NQ-rich stretch encompassing the N-terminal 39 aa (*∆NQ*); the first three (aas 40–74; *∆R1-3*), last 2.5 (aas 75–97; *∆R4-6′*) or all 5.5 (aas 40–97; *∆R1-6′*) oligopeptide repeats; the C-terminal portion of the N domain (aas 98–123, *∆CTN*); or the last 2.5 repeats together with the C-terminal portion (aas 75–123; *∆R4-6′CTN*) (Figure 1D). Each of these deletion alleles was substituted for the original *SUP35* allele on a chromosome of a haploid strain and remained under the control of the endogenous *P_SUP35_* promoter, which was crucial for analyzing prion formation and propagation by respective proteins. In addition, each of respective deletion *SUP35N* derivatives, either alone or in conjunction with *SUP35M*, was also fused to a fluorophore, typically a yellow fluorescent protein (YFP) unless stated otherwise, and placed under the control of copper-inducible *P_CUP1_* on a plasmid (Figure 1D). This enabled us to manipulate protein levels and to visualize the formation of liquid condensates and solid amyloid fibrils using fluorescence microscopy (FM). To avoid a potential impact of full-length Sup35, most FM experiments were performed in the strain with the chromosomal *sup35ΔNM* allele, lacking the Sup35NM-coding region [16].

### 2.2. Effects of the Sup35M Domain on Protein Levels, Prion Formation and Propagation

**Figure 2:**
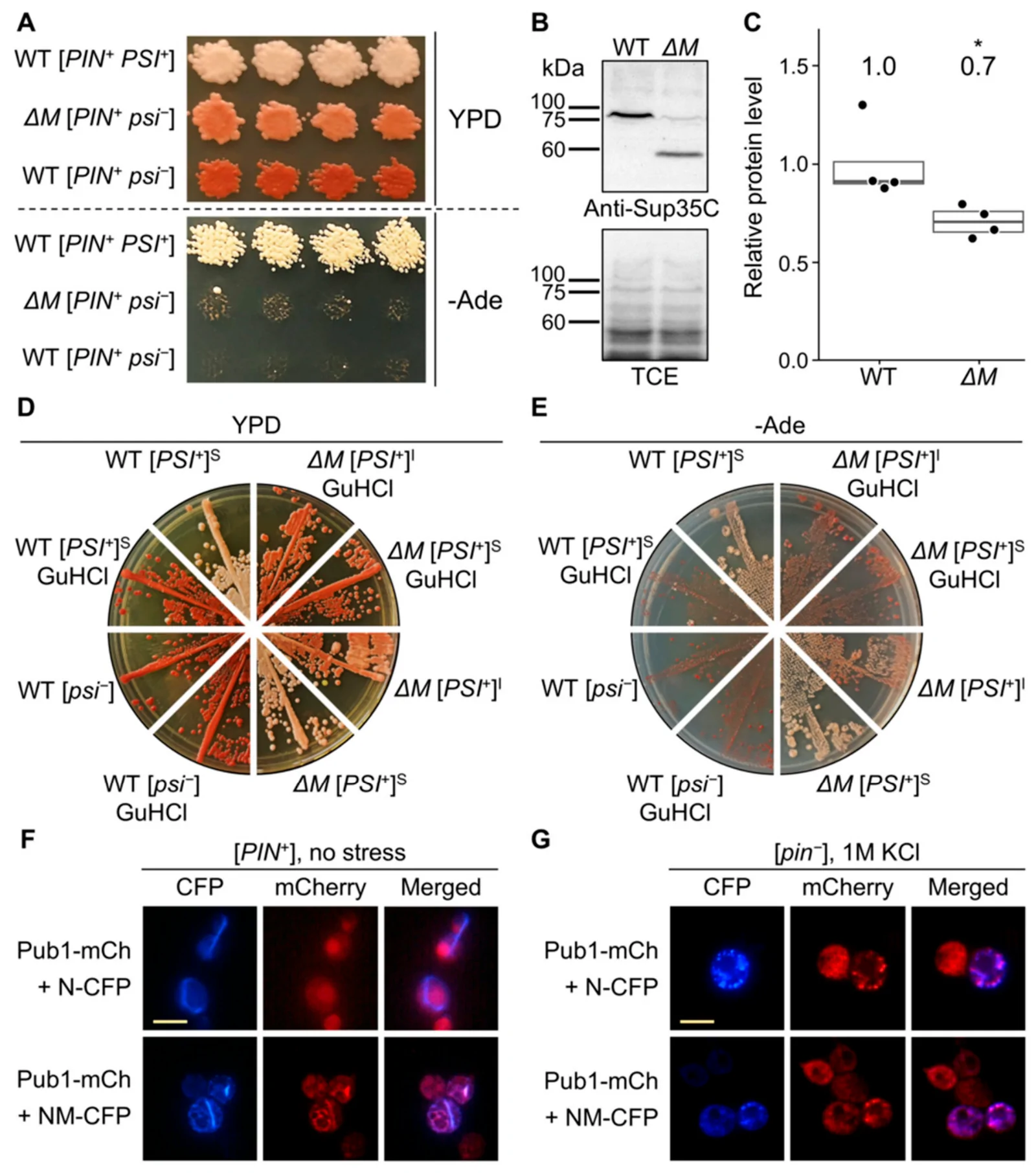
Effects of the Sup35M domain on protein levels, prion properties and interactions with Pub1. (**A**) Deletion of the Sup35M domain reduces accumulation of red pigment on YPD medium and leads to background growth with intense papillation on –Ade medium in a *∆M* [*PIN*^+^ *psi*^−^] strain carrying the *ade1-14* mutation, compared to the isogenic WT [*PIN*^+^ *psi*^−^] strain. (**B**) Deletion of the Sup35M domain decreases the Sup35 protein levels, as shown by Western blotting and reaction to anti-Sup35C antibodies; a representative blot is shown. The same membrane stained with 2,2,2-trichloroethanol (TCE) is provided as a loading control. (**C**) Quantification of normalized Sup35 and Sup35∆M protein levels by densitometry, for 4 experiments represented as the box-and-whisker plot in each case. Boxes indicate the interquartile range, and the horizontal lines denote the median. Individual data points are shown as dots. For numbers, see Table S1. (**D**,**E**) Representative YPD (**D**) and –Ade (**E**) plates showing the results of curing of [*PSI*^+^] colonies, produced by the *sup35ΔM* (*ΔM*) strain on –Ade, by guanidine hydrochloride (GuHCl). Strong WT [*PSI*^+^] ([*PSI*^+^]^S^) and [*psi*^−^] strains served as positive and negative controls, respectively. *∆M* [*PSI*^+^]^S^ and *ΔM* [*PSI*^+^]^I^ are strong and intermediate [*PSI*^+^] variants of the ΔM strain, respectively. (The weak variant is not shown.) (**F**,**G**) Impact of the Sup35M domain on colocalization of Sup35-based amyloid filaments (**F**) and liquid condensates (**G**) with Pub1, as detected by fluorescence microscopy in [*PIN*^+^] (**F**) and [*pin^−^*] strains. Sup35N and NM constructs are tagged with CFP as shown; Pub1-mCh—Pub1 tagged with mCherry. Scale bars correspond to 5 µm. Asterisks indicate statistically significant differences between groups as follows: * *p* < 0.05.

**Figure 3:**
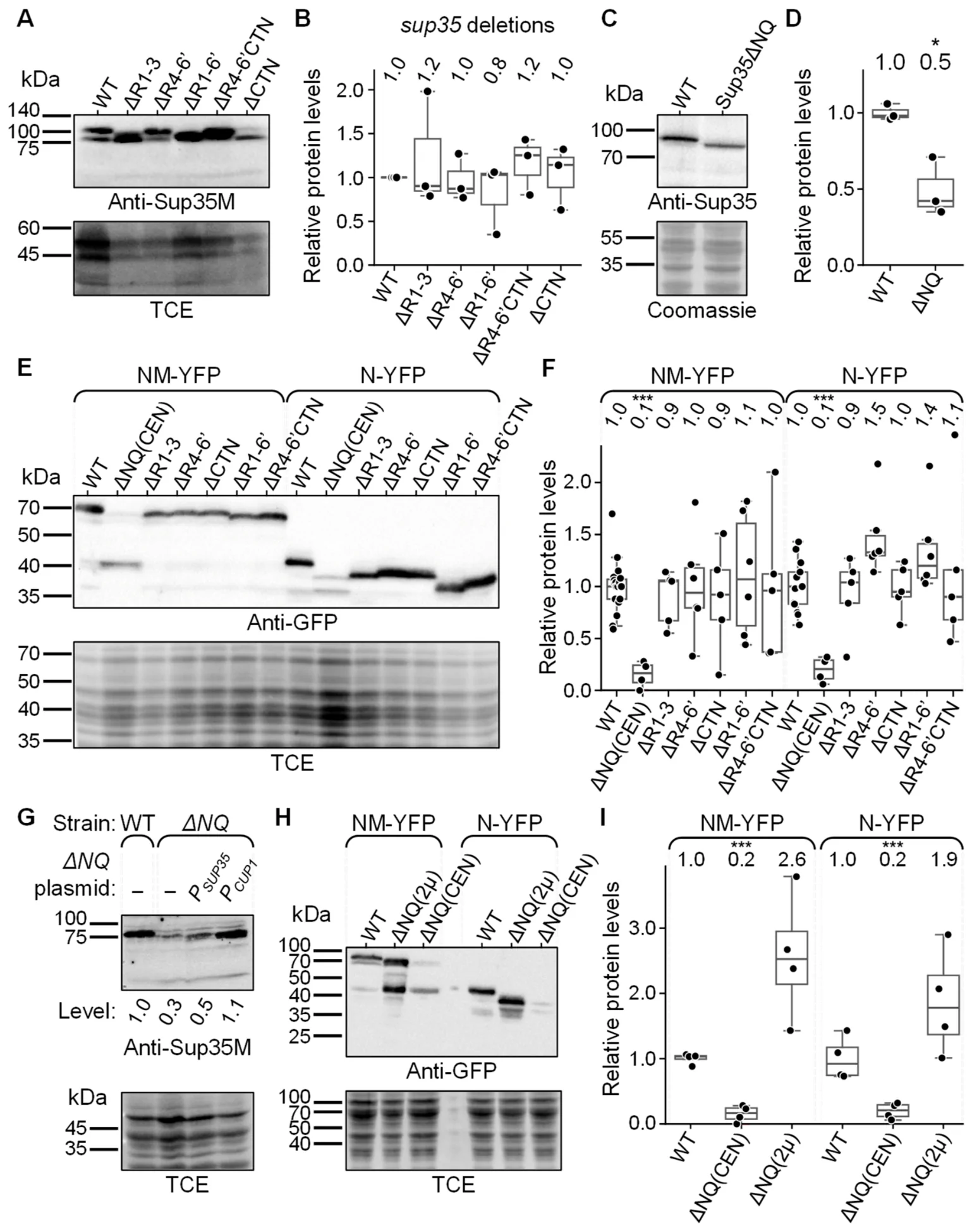
Protein levels of Sup35 deletion derivatives. (**A**–**D**) Representative Western blots (**A**,**C**) and quantification of normalized levels of proteins (**B**,**D**) produced by chromosomal *SUP35* WT and deletion alleles. For numbers, see Table S2 (**B**) and Table S3 (**D**). (**E**,**F**) Representative Western blot and quantification of normalized levels of plasmid-encoded Sup35N-YFP and Sup35NM-YFP derivatives (WT and deletions within the Sup35N region as shown). For numbers, see Table S4. (**G**) Representative Western blot showing levels of the Sup35ΔNQ protein expressed either solely from the chromosomal allele or from the chromosomal allele in combination with additional plasmids carrying an extra copy of *sup35ΔNQ* under the endogenous *P_SUP35_* promoter or under the copper-inducible *P_CUP1_* promoter without addition of extra CuSO_4_. Normalized relative protein levels determined by densitometry are indicated below each lane of the top image. For numbers, see Table S5. (**H**,**I**) Representative Western blot (**H**) and densitometry-based quantification (**I**) showing comparison of the levels of YFP-tagged WT and Sup35N/NMΔNQ proteins expressed from centromeric (CEN) or multicopy (2 µ) plasmids. For numbers, see Table S6. On panels (**A**,**C**,**E**,**G**,**H**) the top gel shows that the nitrocellulose (**A**,**G**,**H**) or PVDF (**C**,**E**) membrane reacted to anti-Sup35M (**A**,**G**), anti-Sup35C (**C**) or anti-GFP (**E**,**H**) antibodies, while the bottom gel shows the same membrane stained with 2,2,2-TCE (**A**,**E**,**G**,**H**) or Coomassie (**C**) as a loading control. On all diagrams (**B**,**D**,**F**,**I**), multiple samples are represented as the box-and-whisker plot in each case; boxes indicate the interquartile range, and the horizontal dashed lines denote the median. Mean values are indicated above the plots. Asterisks indicate statistically significant differences between groups as follows: * *p* < 0.05; *** *p* < 0.001.

Our previous work demonstrated that the Sup35M domain antagonizes the formation of liquid condensates by overproduced Sup35-derived constructs under non-stress conditions and is dispensable for condensate formation under osmotic stress [16]. To address the role of the M domain in prion formation and propagation, we employed the chromosomal *sup35ΔM* allele lacking this region (see above, Figure 1C). The *sup35ΔM* [*psi*^−^] strains (both [*PIN*^+^] and [*pin*^−^]) carrying the *ade1-14* (UGA) reporter allele accumulated red pigment on complete (YPD) medium to a lesser extent compared to isogenic wild-type (WT) [*psi*^−^] strains and exhibited a very weak background growth on the medium lacking adenine (-Ade), while the WT [*psi^−^*] strain did not grow at all (Figure 2A and Figure S1A). This indicates that while the Sup35∆M protein remains functional, leading to premature termination of Ade1 translation, the termination efficiency is slightly decreased, compared to the full-length Sup35 protein. Western blot analysis demonstrated that the Sup35∆M protein is accumulated at a lower level (about 70%) relative to the WT Sup35 protein (Figure 2B,C, and Table S1), which likely explains the slight decrease in translation termination efficiency. To assess prion formation by the Sup35∆M protein, we picked up individual Ade^+^ colonies that arose spontaneously and grew significantly faster than the background *sup35ΔM* [*psi*^−^ *PIN*^+^] culture on −Ade medium and colony-purified them by restreaking on -Ade. In all 12 tested *sup35ΔM* Ade^+^ colonies, the Ade^+^ phenotype was mitotically stable on YPD medium but curable after growth in the presence of 5 mM GuHCl, an inhibitor of the Hsp104 chaperone, which is required for the propagation of yeast prions (Figure 3D,E). This indicates that the Sup35ΔM protein has been converted into a prion ([*PSI*^+^]) state in these colonies. Various *sup35ΔM* [*PSI*^+^] isolates displayed a strong (two isolates), intermediate (eight isolates) or weak (two isolates) Ade^+^ phenotype, as judged by colony color on YPD (Figure 2D) and growth on -Ade (Figure 2E). Thus, the Sup35ΔM protein can form prion variants of different stringencies, including strong variants.

### 2.3. Impact of Sup35M Domain on Association with Pub1/TIA1 Protein

Previously, it has been reported that Pub1/TIA-1, an RNA-binding protein involved in mRNA stabilization and post-transcriptional regulation, colocalizes with the Sup35NM amyloid fibrils [30,31]. We examined the role of the Sup35M domain in the interaction with Pub1. For this purpose, we coexpressed either Sup35N- or Sup35NM-CFP (each induced by 100 µM CuSO_4_ from the copper-regulated *P_CUP1_* promoter) together with mCherry-tagged Pub1, produced from its native promoter in the [*psi^−^ PIN*^+^] strain containing the *sup35ΔNM* deletion in the chromosome. We observed that filamentous amyloids, formed by Sup35NM-CFP in these conditions and representing one of the types of an intermediate in the prion generation pathway [32], showed frequent colocalization with Pub1-mCherry (in 39 out of 52 cells with both CFP and mCherry signals), whereas fibrils formed by Sup35N-CFP exhibited only occasional colocalization (in 15 out of 133 cells) (Figure 2F). As Pub1 is also known to participate in the formation of stress-induced condensates (stress granules), we then tested its colocalization with Sup35N- or Sup35NM-CFP liquid condensates formed in response to osmotic stress (1M KCl, 15–20 min) in a [*psi*^−^ *pin*^−^] *sup35ΔNM* strain. Both condensates formed by Sup35N-CFP (121 out of 131 cells) and by Sup35NM-CFP (88 out of 98) frequently colocalized with Pub1-mCherry *(*Figure 2G). These results indicate that the Sup35M domain enhances binding of Sup35 amyloids to Pub1 but is dispensable for Sup35-Pub1 association in liquid condensates (Figure 2G). Notably, Pub1-mCherry itself did not form condensates after 20 min in 1M KCl in the culture that did not coexpress Sup35 PrD (Figure S1B), suggesting that Sup35 condensates can immobilize Pub1.

### 2.4. Impacts of Deletions Within the Sup35N Domain on Protein Levels

Next, we determined whether deletions within the Sup35N region influence protein levels. For the majority of chromosomal deletion constructs expressed from the endogenous *P_SUP35_* promoter, Western blotting and densitometry showed no significant differences in protein abundance relative to the WT (Figure 3A,B, and Table S2). The only exception was the ΔNQ deletion, which decreased protein abundance by about 2-fold (Figure 3C,D, and Table S3). For fluorophore-tagged Sup35N and Sup35NM constructs induced from the copper-regulated *P_CUP1_* promoter by addition of 100 µM CuSO_4_ for 24 h (~100-fold higher protein levels, relative to endogenous expression, according to our previous data [16]), statistically significant differences in protein abundance between the WT and deletion constructs also were not detected, with the exception of the ΔNQ derivative, which displayed an approximate 10-fold reduction in protein levels (Figure 3E,F, and Table S4).

The introduction of centromeric plasmids carrying an additional copy of the *sup35ΔNQ* allele, either under the native *P_SUP35_* promoter or under the *P_CUP1_* promoter into the strain bearing the *sup35ΔNQ* deletion in the chromosome, increased Sup35ΔNQ protein abundance. However, the only moderately expressed (that is, without the addition of excess CuSO_4_) *P_CUP1_*-based construct produced protein at levels similar to the full-length WT protein (Figure 3G and Table S5). Therefore, we used *sup35ΔNQ* deletion strains carrying an extra copy of *sup35ΔNQ* on a plasmid in the experiments aimed at detection and quantifying [*PSI*^+^] formation and maintenance. Multicopy plasmids bearing *sup35(N/NM)ΔNQ-YFP* constructs under the control of the *P_CUP1_* promoter, induced with 100 µM of CuSO_4_, produced respective proteins at levels that were slightly higher than the levels of respective WT proteins expressed from the centromeric constructs in *sup35ΔNM* cells under the same conditions (Figure 3H,I, and Table S6). Therefore, we employed the multicopy plasmids with ΔNQ constructs in most experiments to make protein levels of all deletion derivatives comparable to each other and to the WT control.

### 2.5. Roles of Various Sup35N Subregions in Formation of Liquid Condensates

**Figure 4:**
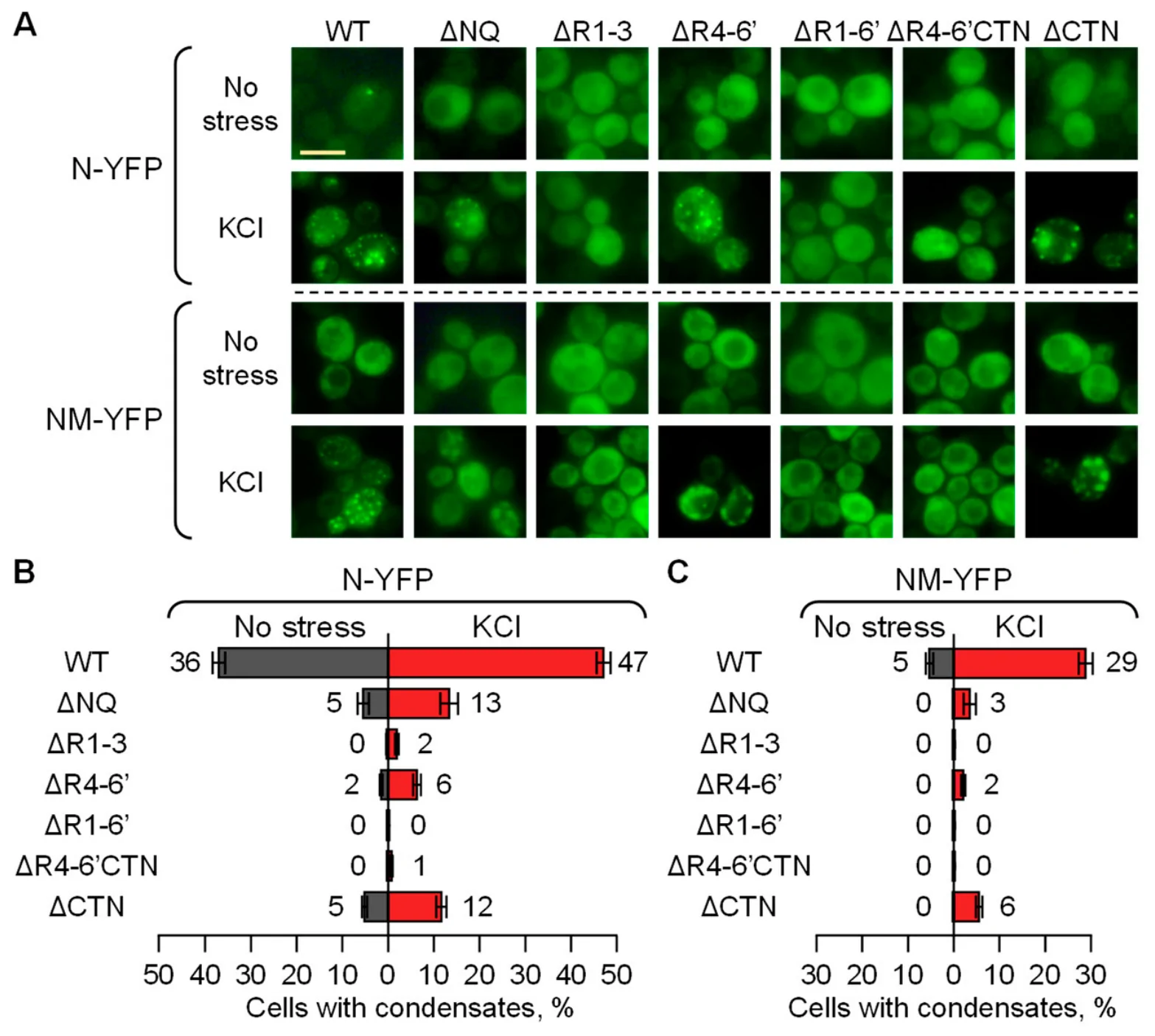
Formation of liquid condensates by YFP-tagged Sup35N/NM derivatives with deletions in the N domain. (**A**) Representative fluorescence microscopy (FM) images. Scale bar corresponds to 10 µm. (**B**,**C**) Quantification of cells with puncta (condensates) for Sup35N-YFP- (**B**) and Sup35NM-YFP-based (**C**) constructs. For panel (**B**), two representative trials are summarized, with one trial including all deletion derivatives except ∆NQ and another trial including only ∆NQ, with WT controls that produced near-identical results between these experiments. For panel C, data of one representative trial is shown. SEs are indicated by error bars. For numbers and results of other trials, see Tables S7 and S8. YFP-tagged constructs were expressed in the [*pin*^−^ *psi*^−^] *sup35∆NM* strain from centromeric plasmids, except ΔNQ, which was expressed from the multicopy (2 μ DNA-based) plasmid. Expression was induced by growth in the presence of 100 µM CuSO_4_ for 24 h. Differences between each deletion derivative and WT control are statistically significant according to Firth penalized binomial logistic regression (*p* < 0.0001).

To assess how various deletions influence the formation of liquid condensates, we overproduced the Sup35N/NM-YFP deletion derivatives in a [*pin*^−^] strain by induction with 100 µM CuSO_4_ and counted cells with condensates before and after osmotic stress (1M KCl). As reported previously [16], overproduced Sup35N-YFP (readily) and Sup35NM-YFP (very rarely) form condensates (visible as small foci) even in the absence of stress, while osmotic stress strongly promotes condensate formation. We found that a deletion of each Sup35N subregion caused a reduction in condensate formation by both Sup35N-based (Figure 4A,B, and Table S7) and Sup35NM-based (Figure 4A,C, and Table S8) constructs, although the magnitude of the effect varied between deletions. The least severe (only several fold) decrease in condensate formation was detected for the ΔNQ (on the 2 µ plasmid) and ΔCTN deletions. On the other hand, deletion of the whole OPR (ΔR1-6′) completely abolished condensate formation in all constructs and conditions tested, and deletion of ΔR4-6′CTN showed a similar loss for the Sup35NM-based construct, with only a slightly higher fraction of cells forming condensates in the stressed culture expressing Sup35N-YFP. Partial OPR deletions showed intermediate effects: ΔR1-3 caused a more severe decrease than ΔR4-6′. As levels of the ΔNQ derivatives expressed from the multicopy (2 µ-based) plasmid were slightly higher than levels of the WT and other deletion derivatives expressed from centromeric plasmids (see above, Figure 3I), we also compared condensate formation by YFP-tagged Sup35NM-based ΔNQ, ΔR1-6′ and ΔR4-6′CTN derivatives when they all were expressed under the induced *P_CUP1_* promoter from the 2 μ-based multicopy plasmids. Once again, the ΔR1-6′ and ΔR4-6′CTN derivatives failed to produce any detectable condensates in both non-stress and stress conditions, while ΔNQ produced some condensates upon osmotic stress (Figure S2). Overall, our data indicates that the OPR is the most crucial determinant of LLPS, with its different parts having quantitatively different impacts.

### 2.6. Roles of Various Sup35N Subregions in the Formation of Filamentous Amyloid Assemblies

**Figure 5:**
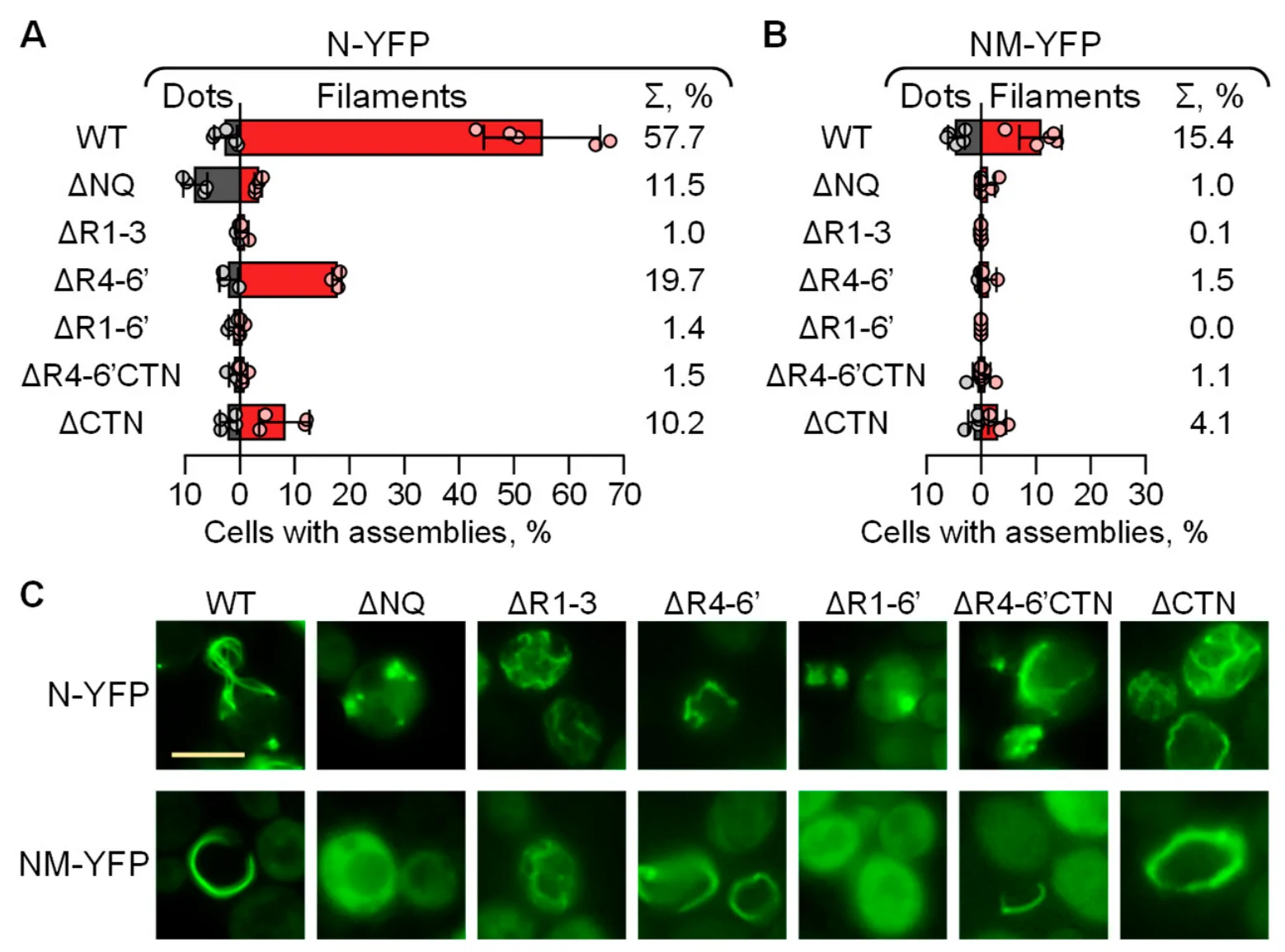
Formation of amyloid assemblies by YFP-tagged Sup35N/NM derivatives with deletions in the N domain. (**A**,**B**) Quantitative data for Sup35N-YFP- (**A**) and Sup35NM-YFP-based (**B**) constructs. On each panel, % of dot-like assemblies is shown by gray boxes on the left and % of filamentous assemblies by the red boxes on the right, with results of individual experiments indicated by circles and SDs indicated by error bars. For numbers, see Table S9. (**C**) Representative FM images. Scale bar corresponds to 10 µm. In all experiments, YFP-tagged constructs were expressed in the [*PIN*^+^ *psi*^−^] *sup35∆NM* strain from centromeric plasmids, except ∆NQ, which was expressed from the multicopy (2 μ DNA-based) plasmid. Expression was induced by growth in the presence of 100 µM CuSO_4_ for 24 h.

As mentioned above, overproduction of fluorophore-tagged Sup35 derivatives in the [*psi*^−^ *PIN*^+^] strain initially produces filamentous amyloid assemblies [33], which represent a predominant species at 24 h of overproduction and correspond to intermediates in the [*PSI*^+^] generation pathway. Punctate amyloid assemblies (dots) also appear and become more abundant with prion maturation. These dots are distinguishable from condensates, detected in [*pin*^−^] cells by their occasionally irregular shape, irreversibility, ability to bind ThT and ThS, and slow fluorescence recovery after photobleaching (FRAP) [16]. We checked the effects of deletions within the Sup35N domain on the formation of amyloid assemblies in [*PIN*^+^] cells. For this purpose, we overproduced deletion variants of Sup35N-YFP and Sup35NM-YFP in the [*psi*^−^ *PIN*^+^] *sup35ΔNM* strain grown in the medium with 100 µM CuSO_4_ for 24 h and monitored aggregate accumulation by fluorescence microscopy. Notably, cells with filaments were more frequent, and the fraction of cells containing only dots was markedly lower in the case of Sup35N-YFP as compared to Sup35NM-YFP (Figure 5A,B, and Table S9). Moreover, cells expressing Sup35N-YFP typically contained multiple intertwined filaments, whereas Sup35NM-YFP yielded 1–2 thick filaments per cell (Figure 5C). These observations indicate that the M domain somewhat counteracts aggregate accumulation and influences aggregate morphology. Deletions within Sup35N impaired amyloid formation, however to different extents (Figure 5). Deletion ΔNQ on the 2 µ-based multicopy plasmid significantly decreased filament formation by both constructs but increased dot formation by Sup35N-YFP. Deletions ΔR4-6′ and ΔCTN had a modest impact on the frequency of cells with filaments (Figure 5A,B). Deletion ΔCTN did not affect filament morphology, while ΔR4-6′ decreased the abundance, length and intertwining of Sup35N-YFP filaments (Figure 5C) and almost completely eliminated dot formation by Sup35NM-YFP (but not by Sup35N-YFP). Deletions ΔR1-3, ΔR1-6′ and ΔR1-6′CTN markedly reduced aggregate abundance, although in the case of Sup35N-YFP, the latter two deletions affected the accumulation of dots less severely than the accumulation of filaments (Figure 5A,B). Notably, both ΔNQ and ΔR1-3 derivatives often produced structurally abnormal assemblies, such as irregularly shaped aggregates, thick and uneven filamentous structures, or wavy filaments (Figure S3). These observations indicate that no single subregion of Sup35N is absolutely required for amyloid formation; however, the cooperative action of several subregions facilitates this process. Notably, the first 74 aas of Sup35N, encompassing the NQ and R1-3 subregions, are important for efficient filament formation and for maintaining correct filament structure, although removal of either subregion does not completely abolish amyloid formation, suggesting partial redundancy.

### 2.7. Roles of Various Sup35N Subregions in the Nucleation of [PSI^+^] Prion

**Figure 6:**
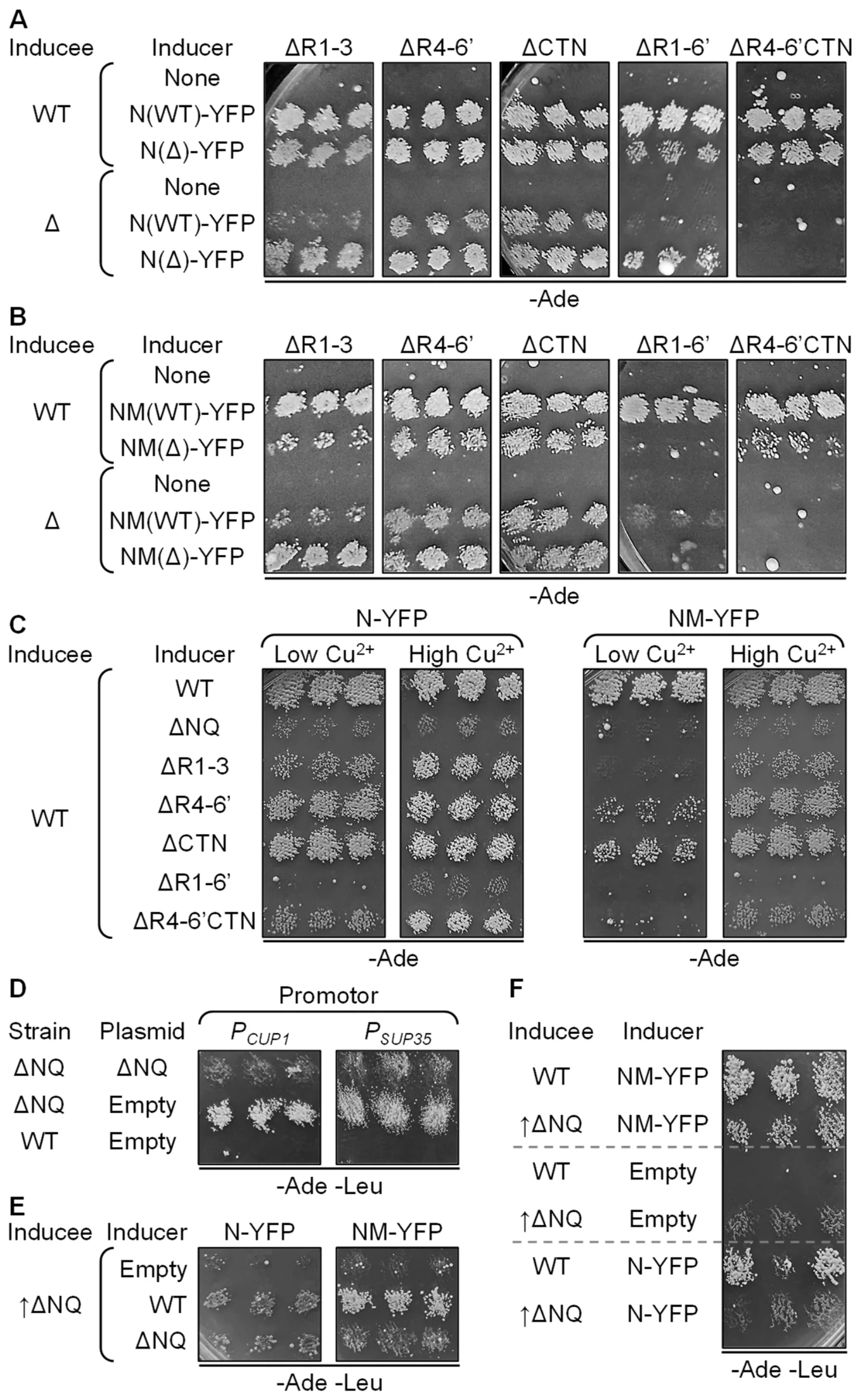
Nucleation of the [*PSI*^+^] prion by Sup35N/NM derivatives with deletions in the N domain. (**A**,**B**) Induction of Ade^+^ colonies indicative of [*PSI*^+^] nucleation by overproduction of Sup35N-YFP (**A**) or Sup35NM-YFP (**B**) inducers (WT or with deletions in the N domain as shown) in [*PIN*^+^ *psi*^−^] inducee strains carrying either full-length wild-type *SUP35* (WT) or *sup35* with a respective deletion (∆) in the chromosome. Transient overproduction of Sup35N/NM derivatives was induced by growth on the medium selective for the plasmid and supplemented by 100 µM CuSO_4_, followed by velveteen replica plating onto the –Ade medium without extra Cu^2+^ for [*PSI*^+^] detection. (**C**) Induction of Ade^+^ formation by Sup35N/NM-YFP derivatives in the WT [*PIN*^+^ *psi*^−^] strain at different levels of overproduction, which are achieved at either background concentration of CuSO_4_, 3 µM (low Cu^2+^, corresponding to moderate protein levels) or with the addition of extra 100 µM CuSO_4_, as in panels (**A**,**B**) (high Cu^2^, corresponding to high protein levels). In both cases, plates were velveteen replica-plated onto the –Ade medium without extra Cu^2+^ for [*PSI*^+^] detection. (**D**) The strain bearing the *sup35∆NQ* allele in the chromosome shows growth on –Ade medium, which is partly compensated by the introduction of the additional copy of the *sup35∆NQ* allele on a centromeric *LEU2* plasmid under the control of either the *P_CUP1_* promoter at background levels of Cu^++^ (**left**) or the endogenous *P_SUP35_* promoter (**right**). (**E**) Induction of Ade^+^ formation by overproduction of WT or ∆NQ derivatives of Sup35N/NM-YFP in the strain carrying the chromosomal *sup35ΔNQ* deletion and *P_CUP1_*-*sup35∆NQ* allele on a plasmid (designated as ↑∆NQ). (**F**) Sup35NM-YFP induces the Ade^+^ phenotype in the strain bearing *sup35ΔNQ* in the chromosome and *P_SUP35_-sup35∆NQ* on a plasmid (↑∆NQ) more efficiently than does Sup35N-YFP. On both panels (**E**,**F**), transient overproduction was induced by extra 100 µM CuSO_4_, while [*PSI*^+^] detection is at background levels of Cu^++^.

Next, we checked whether Sup35N/NM derivatives bearing deletions within the N domain can nucleate formation of the phenotypically detectable [*PSI*^+^] prion. For this purpose, Sup35N-YFP- or Sup35NM-YFP-based constructs on centromeric plasmids (WT or deletion derivatives, with the exception of ΔNQ) were overproduced in the [*psi*^−^ *PIN*^+^] strain carrying either the full-length WT *SUP35* gene or the *SUP35* gene with a respective deletion on the chromosome. This design allowed us to assay both heterotypic (deletion inducer with WT inducee or WT inducer with deletion inducee) and homotypic (deletion inducer with deletion inducee) induction. Formation of a functionally defective (presumably prion) state by a respective chromosomally encoded protein was monitored by readthrough of the *ade1-14* (UGA) allele, detectable on -Ade medium after induction (Figure 6A,B) as described above. All Sup35N-based deletion derivatives were capable of heterotypic Ade^+^ induction in the WT strain, although ΔR1-6′ did so with lower efficiency than other constructs, as judged from the intensity of papillation on -Ade (Figure 6A). This shows that all these deletion derivatives (with a reduced capacity in the case of ΔR1-6′) can nucleate a prion state that templates prion conversion of full-length Sup35. Within the context of a Sup35NM-based construct, ΔR1-6′ almost completely inhibited heterotypic prion induction, while ΔR1-3, ΔR4-6′CTN, and to a lesser extent ΔR4-6′ exhibited a significant reduction in the efficiency of prion induction (Figure 6B), which became more evident when the concentration of CuSO_4_ in the medium (and the level of the inducer protein) was decreased (Figure 6C). Notably, ΔR4-6′CTN derivatives did not show homotypic prion induction; WT Sup35N- or Sup35NM-YFP also failed in inducing the Sup35ΔR4-6′CTN protein into a prion, suggesting that this derivative cannot maintain a prion state (Figure 6A,B). The Sup35-ΔR1-6′ protein also was not induced into a prion state after overproduction of WT Sup35N- or Sup35NM-YFP or of Sup35NM-ΔR1-6′-YFP (Figure 6A,B); however, low efficient homotypic prion induction by Sup35NΔR1-6′-YFP was detected (Figure 6A). Other deletion derivatives were induced into a prion state by both the homotypic inducer and WT Sup35N- or NM-YFP, with homotypic induction being more efficient than heterotypic induction by the WT inducer for at least ΔR1-3 and ΔR1-6′ (Figure 6A,B).

As deletion ΔNQ decreases protein levels (see above, Figure 3), we employed the multicopy (2 µ DNA-based) plasmid for the heterotypic induction experiments. Heterotypic induction of the Ade^+^ phenotype by multicopy plasmids encoding Sup35NΔNQ-YFP or Sup35NMΔNQ-YFP was detected, although with lower efficiencies as compared to other deletion derivatives except ΔR1-6′ (Figure 6C). Homotypic induction experiments with ΔNQ were complicated by the fact that decreased levels of the Sup35ΔNQ protein (when produced from a chromosomal gene, see above, Figure 3C,D) resulted in the partial defect of translation termination, leading to high background growth on -Ade medium that interferes with phenotypic detection of [*PSI*^+^] (Figure 6D). To reduce background growth, we introduced an additional centromeric *LEU2* plasmid carrying the *sup35ΔNQ* allele into the strain bearing the chromosomal *sup35ΔNQ* allele and monitored the phenotype on -Ade -Leu medium that was selective for the plasmid. The extra copy reduced background growth on -Ade when driven by the *P_SUP35_* promoter and almost completely abolished growth when driven by the *P_CUP1_* promoter (Figure 6D), consistent with partial and complete restoration of Sup35ΔNQ protein levels in these cases, respectively (Figure 3I). After resolving the background growth issue, induction of Ade^+^ by both Sup35N- or Sup35NM-YFP and by respective homotypic ΔNQ constructs was detected in strains expressing the Sup35ΔNQ protein from both chromosomal and plasmid-borne alleles simultaneously (Figure 6E). In contrast to some other deletions, Sup35NM-YFP induced conversion of the Sup35ΔNQ protein into a prion state more efficiently than Sup35N-YFP (Figure 6F). Overall, our data confirms that contrary to previous reports [23,24], the Sup35 derivatives lacking the NQ region can both nucleate prions and be induced into a prion state in yeast cells.

### 2.8. Roles of Various Sup35N Subregions in the Propagation of the [PSI^+^] Prion

**Figure 7:**
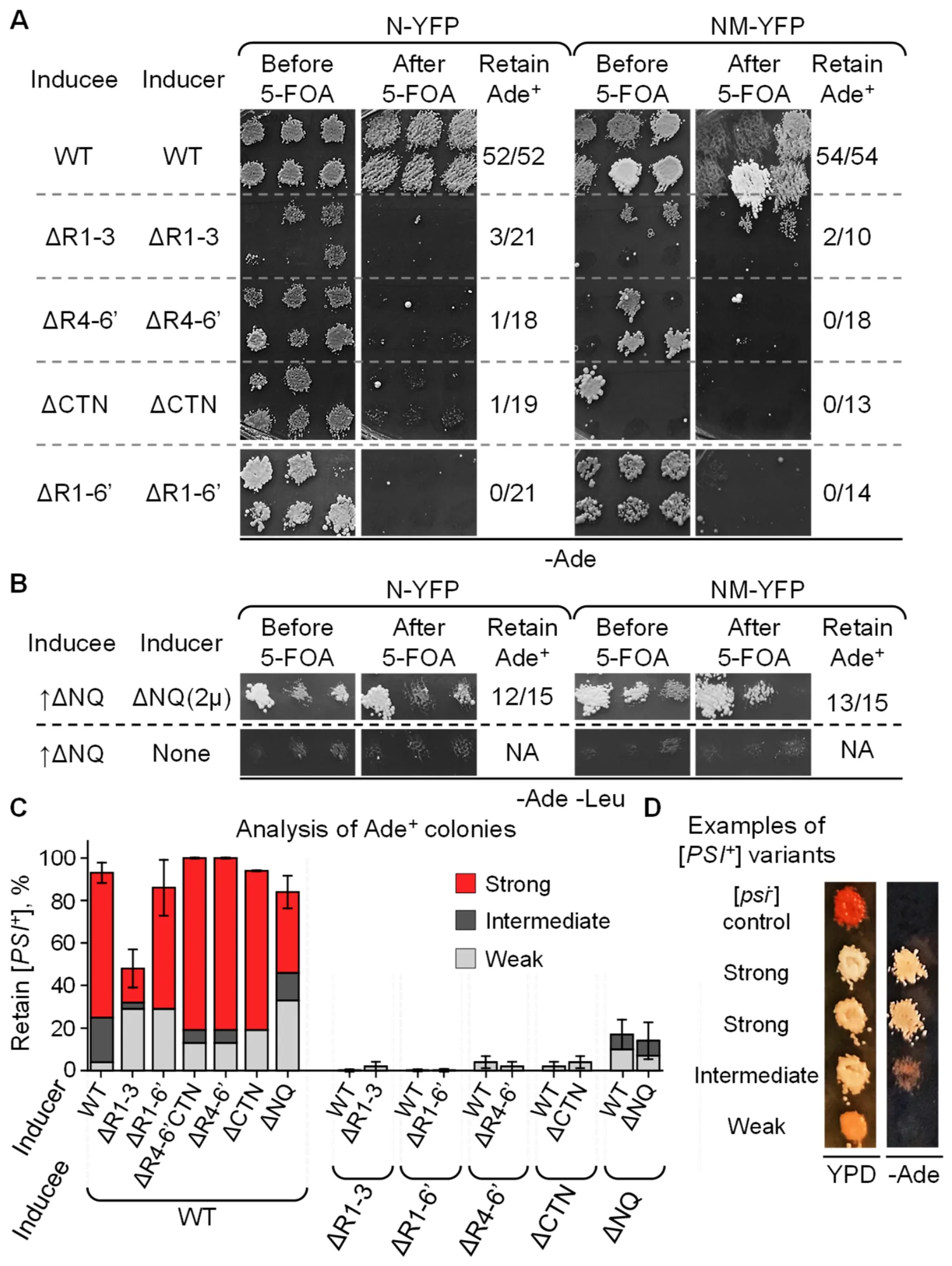
Propagation and heritability of the [*PSI*^+^] prion produced by Sup35N/NM derivatives with deletions in the N domain. (**A**,**B**) Retention of the Ade^+^ phenotype after removal of inducing plasmids. Ade^+^ derivatives of WT or *sup35∆* strains induced by Sup35N-YFP (on the left) or Sup35NM-YFP (on the right) deletion variants were plated on −Ade before and after counterselection on 5-fluoroorotic acid (5-FOA), eliminating the inducer *URA3* plasmids. Growth on -Ade after 5-FOA reflects retention of the induced [*PSI*^+^] state. Numbers on the right of images indicate the number of colonies that retained the Ade^+^ phenotype among all colonies tested after 5-FOA. On panel B, retention of the Ade^+^ phenotype is tested in the *sup35∆NQ* strain bearing an additional copy of *sup35∆NQ* on a *LEU2* plasmid under the *P_CUP1_* promoter (↑∆NQ) in order to equalize protein levels and decrease background growth on the [*psi^−^*] strain on -Ade medium (see Figure 6D). The non-induced ↑∆NQ control is shown at the bottom. (**C**) Quantitative analysis of [*PSI*^+^] inheritance in mitotic divisions in the induced Ade^+^ colonies, which were colony-purified after 5-FOA treatment (shown on panels (**A**,**B**)) and incubated through three passages on YPD medium. Curability of the Ade^+^ phenotype by GuHCl has been confirmed in parallel, as shown in Figure S3. For those retaining [*PSI*^+^], proportions of strong, intermediate and weak [*PSI*^+^] variants are indicated respectively by red, dark grey and light grey boxes as shown. Cumulative data for Sup35N- and Sup35NM-based inducers are shown. See Materials and Methods for details. For numbers, see Tables S10–S13. (**D**) Examples of [*PSI*^+^] variants. Images show representative colonies of the WT strain induced by Sup35NΔR1-3. Strong [*PSI*^+^] variants are whitish on YPD and grow strongly on −Ade, whereas weak [*PSI*^+^] variants are pink on YPD and grow weakly on −Ade. Intermediate [*PSI*^+^] variants show an intermediate phenotype.

To assess the propagation and heritability of the prion state nucleated and/or maintained by deletion derivatives of Sup35, we checked the ability of Ade^+^ colonies, induced as described above in the Section 2.7, to maintain the Ade^+^ phenotype after the loss of the inducer plasmid (coding for Sup35N-YFP or Sup35NM-YFP or their derivatives carrying respective deletions, depending on the sample). Plasmids were removed by growth on 5-fluoroorotic acid (5-FOA), which counterselects against cells carrying the plasmid marker *URA3* [34]. In contrast to prions formed by full-length Sup35, which are typically maintained independently of the presence of the inducing plasmid, the Ade^+^ phenotype was preferentially or completely lost upon plasmid removal in all strains expressing the chromosome-encoded Sup35 deletion derivatives (Figure 7A). The exceptions from this conclusion were the *sup35ΔR4-6′CTN* strain, which does not acquire the Ade^+^ phenotype at all (see Figure 6B,C), and the *sup35ΔNQ* strain, in which the Ade^+^ phenotype, detectable in the presence of an extra copy of the *sup35ΔNQ* gene, as described above, has been retained after loss of the inducer construct (Figure 7B).

At the next step, derivatives retaining the Ade^+^ phenotype were colony-purified and analyzed for phenotypic stringency, mitotic stability, and curability by GuHCl, an agent eliminating the Hsp104-dependent yeast prions including [*PSI*^+^] [35] (Figure S4). As expected, the WT strain typically produced relatively stable, GuHCl-curable [*PSI*^+^] prions of various stringencies, defined as strong, intermediate and weak variants based on the intensity of growth on −Ade and extent of losing red coloration on YPD (Figure 7C,D, and Tables S10 and S11). However, the spectrum of Ade^+^ isolates formed by WT Sup35 was influenced by an inducer protein. Strong prions prevailed with a homotypic WT inducer, while weak strains prevailed with of ΔR1-3 and Sup35NΔNQ inducers, and the ΔR1-3 inducer (especially in the Sup35NM context) produced the largest proportion of highly unstable isolates (Figure 7C, and Tables S10 and S11). This shows that properties of the initial nucleus can influence features of a resulting prion.

In agreement with qualitative assessment (Figure 7A), most Ade^+^ colonies produced by inducee proteins with deletions were mitotically unstable and almost completely lost the Ade^+^ phenotype in non-selective conditions, indicating a defect in prion propagation (Table S12). Occasional GuHCl-curable [*PSI*^+^] strains were detected only in isolated cases and usually were phenotypically weak. Importantly, the *sup35ΔR1-6* derivative did not yield any GuHCl-curable Ade^+^ colonies, suggesting that this derivative is completely unable to propagate the prion state. A notable exception, also agreeing with qualitative data, was the *sup35ΔNQ* derivative that produced a substantial fraction of relatively stable, GuHCl-curable [*PSI*^+^] isolates of weak or intermediate phenotypic stringencies (Figure 7C and Table S10). This indicates that Sup35ΔNQ prions are capable of Hsp104-mediated fragmentation and propagation, although at lower efficiency as compared to prions formed by full-length Sup35.

## 3. Discussion

### 3.1. Sup35M Antagonizes LLPS and Amyloid Formation and Is Dispensable for Prion Propagation

The literature data point to the role of the Sup35M domain as a pH sensor in the modulation of Sup35 LLPS, induced by acidic stress, although this interpretation has been questioned by another paper [15]. In combination with our previous data [16], our new findings indicate that the presence of the Sup35M domain counteracts both LLPS and spontaneous Sup35 prion conversion. Sup35NM-based constructs were consistently less efficient in all assays for condensate (Figure 4B,C) and amyloid (Figure 5A,B) formation compared to Sup35N-based constructs. Nucleation of the full-length Sup35 protein into the [*PSI*^+^] prion after heterotypic induction by Sup35NM-based constructs carrying various deletions was also less efficient than by Sup35N-based constructs, which is especially evident at low levels of overproduction, that is, in the absence of excess CuSO_4_ (Figure 6C). One notable exception was a Sup35NMΔNQ derivative, which induced prion formation by a homotypic Sup35ΔNQ inducee more efficiently as compared to the Sup35NΔNQ inducer (Figure 6F). This could be due to the fact that a significant portion of the amyloidogenic region is lost in the ΔNQ region, leading to the activation of secondary amyloidogenic stretch, as described in vitro for the mutation within the NQ region [36]. Otherwise, our data are consistent with the M domain maintaining Sup35 in a soluble functional state. Perhaps charged residues of Sup35M producing repulsive interactions play a role in this process, which may explain why neutralization of the charge at an acidic pH could increase LLPS in these conditions [14]. Ade^+^ colonies with [*PSI*^+^] properties spontaneously appeared more frequently in the [*psi*^−^ *PIN*^+^] *sup35ΔM* culture as compared to the WT culture (Figure 2A). However, accurate quantitative comparison was complicated due to the high background growth of the *sup35ΔM* strain on –Ade medium. We found that deletion of the M domain caused a modest reduction in protein abundance (Figure 2B,C), which explains the reduction of translation termination, leading to background growth on -Ade.

Previous data indicated that first 114 aas of Sup35N are sufficient to transmit the [*PSI*^+^] prion in cytoduction experiments [37], although later studies indicated that [*PSI*^+^] variants formed by the Sup35 ΔM derivative are characterized by reduced solubility and decreased mitotic stability of a prion, suggesting the role of the M domain in [*PSI*^+^] propagation [38]. Notably, the aa 129–148 region is needed for [*PSI*^+^] curing by overproduction of the *Hsp104* chaperone, thus pointing to the role of the M domain in interactions with chaperones, even though this domain is not required for prion propagation [39]. Indeed, binding of Hsp104 to the Sup35 region including the end of N domain and beginning of M domain (96–151) has been reported [40]. More recently, Shen et al. [41] argued that the overlapping Ssa1/Hsp70-binding site between residues T143 andK164 promotes Sup35 amyloid disaggregation, involved in prion propagation. However, we found that the [*PIN*^+^ *psi*^−^] *sup35ΔM* culture yielded colonies that could maintain a stable heritable [*PSI*^+^] phenotype and were curable by GuHCl, an inhibitor of Hsp104 (Figure 2C,D). Thus, our results clearly show that stable [*PSI*^+^] variants can arise spontaneously in the absence of Sup35M and remain dependent on the chaperone machinery.

### 3.2. Role of Sup35M in Interactions of Liquid and Solid Sup35 Assemblies with Other Proteins

Previous data [31] also pointed to the role of the Sup35M domain in interaction with TIA1, the human ortholog of the yeast Pub1 protein, which is involved in the assembly of stress granules, stress-induced cytoplasmic condensates containing translationally repressed mRNAs, RNA-binding proteins, and components of translation machinery [42,43]. The formation of canonical stress granules in yeast cells is weak or undetectable during osmotic stress, consistent with our observation that yeast Pub1 tagged with mCherry did not form detectable condensates in osmotically stressed yeast cells expressing Sup35 without the NM region. However, overproduction of Sup35N or Sup35NM tagged with CFP recruited Pub1 into liquid condensates (Figure 2G). Thus, Sup35N is sufficient for Pub1 recruitment, while Sup35M is not needed for this process. This data implicates Sup35N as a potential scaffold capable of recruiting other proteins into liquid condensates in conditions when Pub1 cannot nucleate granules on its own. Notably, amyloid filaments assembled from fluorophore-tagged Sup35 derivatives in the process of [*PSI*^+^] formation in [*PIN*^+^] cells also recruited Pub1 (Figure 2F); however, in this case, Pub1 recruitment by Sup35NM filaments was significantly more frequent than by Sup35N filaments. It is likely that Pub1 is interacting with the portion of Sup35 IDR that is not included in the amyloid core and is exposed on the side of the fibril; such a portion would be expected to become significantly larger if Sup35M is present.

### 3.3. Overlapping but Distinct Roles of Sup35N Subregions in the Formation of Liquid and Solid Assemblies

Our deletion analysis reveals overlaps in Sup35N subregions involved in the formation of liquid condensates and amyloid fibrils. We found that the OPR plays a major role in both processes, while deletions of NQ and especially CTN regions had significantly less pronounced effects (Figure 4 and Figure 5). Notably, deletion of the second half of this region (ΔR4-6′) showed a significantly weaker effect compared to the deletion of the first half (ΔR1-3) or especially the whole region (ΔR1-6′), suggesting that either the length of the repeat region is important (the R1-3 region is longer than R4-6′ by 12 aas) or the first half of the OPR contains some sequence features that make it more important than the second half. However, the combination of ΔR4-6′ with ΔCTN made the effect severe, even though ΔCTN alone exhibited only a moderate impact. One possible explanation for this is that a decrease in length of the Sup35N region (that is most significant in the ΔR1-6′ and ΔR4-6′CTN derivatives) decreases the efficiency of intermolecular interactions, leading to the formation of both liquid and solid assemblies. However, length is definitely not the only factor influencing these processes, as the ΔNQ derivative, which is slightly shorter than ΔR1-3, and the ΔCTN derivative, which is slightly shorter than ΔR4-6′, show less severe defects. It is therefore likely that liquid and solid transitions are mediated by partially redundant sequence elements acting in a cooperative fashion. Similarity in the effects of one and the same deletion on liquid and solid transitions suggests that these processes could be linked. This is also supported by previous observations by us [16] and others [44] that osmotic stress enhances [*PSI*^+^] induction in [*PIN*^+^] cells. However, LLPS of *S. cerevisiae* Sup35 in [*pin*^−^] cells is not sufficient for amyloid formation [16]. Thus, Sup35 LLPS may create conditions favorable for amyloid conversion, but efficient transition to amyloid would require an additional factor such as Rnq1 prion. Alternatively, it is possible that LLPS and amyloid formation involve interactions between overlapping sequences that lead to different outcomes.

The ability of most deletions to nucleate prion formation by the full-length Sup35 prion (Figure 6) agrees with previous observations indicating that presumptive amyloid core regions may extend beyond a single sequence element, such as NQ, OPR or CTN [45,46], as well as with the potential existence of secondary amyloidogenic stretches within Sup35N [36], which could be activated if formation of a major core is impaired. It remains to be determined whether amyloid core composition is retained or altered after the amyloid state is transferred from truncated to full-length PrD. Analysis of the spectra of prion variants indicates that it is influenced by both inducer and inducee proteins, with further investigations needed for deciphering mechanisms of their impacts.

### 3.4. Dispensability of Sup35N NQ-Rich Stretch for Sup35 Amyloid Formation and Prion Nucleation

Previous studies identified the amino-terminal NQ-rich stretch as critical for Sup35 amyloid formation and prion induction in yeast [23,24]. This contradicted results in mammalian cells where this region was dispensable for the aggregation of heterologous yeast Sup35NM [47]. Our data eliminate this contradiction by showing that ΔNQ deletion reduced but did not abolish Sup35 LLPS (Figure 4), amyloid formation (Figure 5) and prion nucleation (Figure 6C–F). The discrepancies with previous studies may be explained, at least in part, by the fact that ΔNQ strongly reduces protein abundance (Figure 3C–I), the effect of which we were able to compensate for by using multicopy constructs. It is not likely that protein destabilization is simply due to the generation of a destabilizing sequence at the N-terminus, because our plasmid-borne ΔNQ constructs retained the second Sup35N residue, S (in addition to initiator M), but this did not restore protein abundance. It has been proposed that the NQ-rich composition of N-proximal regions protects PrDs from degradation [48], suggesting that the NQ-rich region may contribute not only to Sup35 assembly but also to Sup35 proteolytic stability. Dispensability of the NQ-rich stretch for amyloid formation agrees with the fact that the NQ contents of Sup35N remain high even after deletion of the N-proximal NQ stretch and with previous observations indicating that the amyloid core protected from hydrogen–deuterium exchange may continue beyond this stretch, especially in weaker prion variants [45].

### 3.5. Sequence Requirements for Prion Nucleation and Inheritance Are Different from Each Other

[*PSI*^+^] propagation and inheritance require not only the initial formation of Sup35 amyloid aggregates but also the generation of a sufficient number of transmissible prion seeds via chaperone-mediated amyloid fragmentation, thus allowing repetitive cycles of fibril growth by Sup35 recruitment and achieving prion transmission in cell divisions [19,49,50]. A significant fraction of Sup35 aggregates produced upon overproduction cause a transient decrease in translation termination, which is phenotypically detectable as nonsense suppression [51] but fails to establish a stable heritable [*PSI*^+^] state [52].

Previous data indicated that progressive shortening of the Sup35 OPR decreased [*PSI*^+^] heritability [25], with a minimal region required for [*PSI*^+^] maintenance being defined between 1–64 and 1–83, depending on the prion strain [27]. No impact of the deletion 98–112 on [*PSI*^+^] inheritance has been detected [25]. The downsides of these experiments were that they only looked at the maintenance of one or several pre-existing [*PSI*^+^] variants, initially obtained in the WT Sup35 background, and employed plasmid-borne *SUP35* that is expressed at higher levels compared to the chromosomal allele, even when located at a centromeric plasmid under the endogenous *P_SUP35_* promoter [16]. The paper by Langlois et al. [26] expanded this analysis using the integrated chromosomal alleles and confirmed that progressive shortening of the OPR decreases [*PSI*^+^] heritability, with constructs bearing three or less repeats being unable to maintain the prion. The authors also found that both repeats and the region 97–111 modulate fragmentation of the Sup35 polymers by the chaperone machinery. However, these experiments were also performed in specific strains with a pre-existing [*PSI*^+^] prion. Moreover, none of these works specifically addressed the impact of the whole CTN region, as the fragment after position 112/113 was initially considered to be dispensable for [*PSI*^+^] maintenance [22], even though it was later found to be needed for the transmission of weak variants of [*PSI*^+^] [53].

Investigation of the impact of deletions on the inheritance of only specific prion variants may unintentionally uncover variant-specific effects, as described previously for the point mutation PNM2 (G58D) [54,55]. To avoid such a possibility, we have performed a systematic analysis of the impacts of Sup35N regions on both initial prion nucleation and prion heritability. To minimize variant-specific impacts, we analyzed multiple prion isolates, nucleated *de novo* by either WT PrD or PrDs with deletions on a full-length recipient (inducee) protein with or without deletion, that were encoded by a respective chromosome allele. It is likely that due to their altered sequences, deletion derivatives produce prion variants with different conformational properties, which alter interactions with chaperone machinery as compared to the full-length protein. Therefore, these differences would reflect different sequence requirements for chaperone-mediated fragmentation, allowing us to decipher the impact of specific sequences on this process. The comparison between the full-length and deletion inducees enables us to differentiate impacts of an initial seed versus a substrate protein. We believe that our approach provides a more comprehensive and less biased picture as compared to previous studies employing specific prion variants.

All deletion derivatives of Sup35N retained the ability to nucleate the prion state at least in some constructs and/or inducer/inducee combinations, although some of them did so with a decreased efficiency (Figure 6A,B). Notably, heterotypic [*PSI*^+^] nucleation by deletion derivatives on the WT Sup35 inducee was typically better detectable than homotypic nucleation using the same derivatives, to the extent that a derivative lacking the last three repeats and CTN region (ΔR4-6′CTN) was unable to nucleate a detectable prion in a homotypic combination at all (Figure 6A,B). This partly correlated with the reduced formation of amyloid fibrils by most deletion derivatives (Figure 5A,B). In contrast, prion nucleation by a homotypic inducer was more efficient than nucleation by a heterotypic WT inducer at least for deletion derivatives missing the first portion (ΔR1-3) or the whole (ΔR1-6′) OPR (Figure 6A,B). This may reflect the fact that the initial portion of the OPR could be included in the amyloid core formed by the WT inducer [45,46]. This observation indicates that deletions may alter the local context of neighboring regions and create partial transmission barriers, in agreement with previous data showing that even closely related Sup35 PrDs, capable of coaggregating, may exhibit a decreased cross-species transmission of the prion state, possibly due to critical amyloid-forming or recognition elements being partially mismatched [56,57,58,59]. However, the relationship between heterotypic and homotypic induction has been reversed for the ΔNQ inducee, perhaps due to a decreased capability of forming the initial amyloid core in the respective homotypic inducer protein (Figure 6E).

In addition, our data show that all [*PSI*^+^] variants produced by deletion derivatives, with the exception of ΔNQ, were highly unstable and typically quickly lost in cell divisions after the loss of the inducer plasmid (Figure 7 and Tables S10–S12). Surprisingly, this also applied to ΔCTN. As 97/98–111/112 deletions have not previously been reported to completely destabilize prion inheritance (see above), this result signifies the importance of the 113–125 region, which did not attract significant attention in most of the previous studies. Even in the case of the WT inducee, deletion derivatives typically induced a larger proportion of weak prion variants compared to a homotypic WT inducer (Figure 7C). This indicates that deletion derivatives tend to form amyloid fibrils that are less efficiently fragmented by a chaperone machinery as compared to the fibrils formed by full-length WT protein, and these features are apparently determined by patterns of an amyloid core that are at least partially reproduced in the full-length fibrils acquiring the prion state from deletion derivatives.

Notably, ΔR4-6′CTN (that is, C-terminal deletion of Sup35N) and ΔNQ (N-terminal deletion) derivatives exhibited differential relationships between amyloid formation, heterotypic prion nucleation of WT Sup35, and propagation of a heritable prion. ΔR4-6′CTN formed amyloid filaments inefficiently (Figure 5A,B) and failed to establish a detectable [*PSI*^+^] prion (Figure 6A,B) but nucleated full-length WT Sup35 into [*PSI*^+^] relatively efficiently, especially when overproduced within the context of the Sup35N-based construct (Figure 6A–C). These data agree with previous reports indicating that the major amyloid core protected from hydrogen–deuterium exchange [45] or proteolysis [46] is typically confined within the first 70 aas of Sup35N that are retained in the ΔR4-6′CTN derivative. In contrast, ΔNQ formed amyloid aggregates more efficiently than ΔR4-6′CTN (Figure 5A,B) but nucleated full-length WT Sup35 into [*PSI*^+^] poorly (Figure 6C), consistent with missing a portion of the potential WT core. It is possible that in the ΔNQ derivative, the core is shifted towards the C-end of the N-domain, as suggested previously from the analysis of an anti-aggregation mutant, also located within the NQ stretch [36]. In contrast to other deletion derivatives, the Sup35NM-based construct nucleated the Sup35ΔNQ protein into [*PSI*^+^] state more efficientlly, as compared to the Sup35N-based construct (Figure 6A–C,F). It is therefore possible that the shifted amyloidogenic core activated in case of the deletion of the QN stretch includes the beginning portion of the Sup35M domain. However, ΔNQ was the only deletion derivative that retained a significant fraction of heritable [*PSI*^+^] isolates after the loss of plasmids, even though they were all intermediate or weak, and their mitotic stability was reduced in comparison to the WT (Figure 7B,C). This agrees with the previously proposed role of the OPR and CTN in chaperone binding and fibril fragmentation, although it is likely that the shifting of an amyloid core makes a portion of this region poorly accessible for the chaperone machinery, thus somewhat impairing but not completely eliminating prion propagation.

### 3.6. Composition-Based Versus Modular Organization of Sup35 PrD

Previous publications provided evidence in favor of both the modular organization of Sup35 PrD [24,25,26] and aa-composition-dependent organization, in which the existence of defined modules would be hard to imagine [28,29]. While our data do not uncover a set of independent functional modules within Sup35N, they point to differential though partly overlapping and redundant roles of different subregions, making the composition-based model oversimplified. Rather, the intact N-domain functions as an integrated prion-forming unit in which the NQ, OPR, and CTN regions make distinct but cooperative contributions to amyloid nucleation, amyloid growth, conformational compatibility, chaperone-dependent processing, and stable [*PSI*^+^] inheritance. While deletions of individual regions do not necessarily completely abolish phase separation, amyloid aggregation or prion nucleation, they reduce the efficiencies of these processes to various extents. Each tested deletion significantly compromised prion inheritance, with ΔNQ having the least pronounced impact compared to others.

How can the differential impact of specific subregions be reconciled with the prion-forming abilities of scrambled Sup35 PrD derivatives described previously [28]? To explain this, one should take into account the low complexity of the Sup35N sequence. Due to the high abundance of some aa residues (such as Q, N, Y, etc.) and low abundance of others (for example, charged residues), some sequence motifs or signatures that are important for amyloid formation and/or propagation could be reconstituted with a high probability after reshuffling. The importance of oligopeptide repeats could be in achieving the periodical distribution of some residues included in these repeats. It has been reported, for example, that not only abundance but periodical spacing of aromatic residues, which serve as “stickers” separated by spacers, could play a major role in LLPS [60,61], and Ys are indeed included in the repeated units. Likewise, interspacing of Qs with Ys promotes the fragmentation of artificially generated polyQ amyloids in yeast cells [62]. In such a case, a pattern important for a given process could be occasionally (and if particular aa residues are abundant, even frequently) reconstituted after the random sequence reshuffling, even though an overall sequence of the respective region is not maintained. Further research is needed to address these issues.

Overall, our data show that despite seeming to have low complexity, Sup35 IDR has a hierarchical architecture, so that both aa composition and specific sequence arrangements play roles in modulating its phase separation, aggregation and prion properties.

## 4. Materials and Methods

### 4.1. Media and Cultivation Conditions

Standard yeast cultivation procedures and media were described previously [63]. Rich medium (YPD) contained 1% yeast extract, 2% peptone, and 2% dextrose. YPG medium had the same composition except that it contained 2% glycerol instead of dextrose. Synthetic medium contained 0.67% yeast nitrogen base without amino acids and ammonium sulfate, 0.5% ammonium sulfate, 2% dextrose, and a supplement mixture of 13 nutrients (adenine, arginine, histidine, isoleucine, leucine, lysine, methionine, phenylalanine, threonine, tryptophan, tyrosine, uracil, and valine), unless specific components were omitted as indicated (e.g., –Ade for medium lacking adenine). The standard synthetic medium contains approximately 3 µM Cu^2+^, which permits basal expression from the *P_CUP1_* promoter. Overexpression was induced by addition of 100 µM CuSO_4_, a concentration that is non-toxic for most laboratory *S. cerevisiae* strains [64], including those used in this study. As our strains contained a mutation in the *ADE1* gene, extra adenine was added to the growth medium for cultures used in FM at a final concentration of 200 mg/L to prevent accumulation of the red pigment, polymerized from an intermediate of adenine biosynthesis. The [*PSI*^+^] prion was monitored by growth on –Ade medium and by lighter color on YPD or ¼YPD (containing only 0.25% of yeast extract rather than 1%) due to readthrough of the reporter *ade1-14* (UGA) allele, as described previously [65]. Yeast cultures were grown at 30 °C unless otherwise specified. Due to slow growth of some [*PSI*^+^] strains in selective conditions, –Ade plates were typically scanned after 10–14 days of incubation. Color on YPD plates was scored after 2–3 days of incubation, sometimes followed by storage in a refrigerator for several days that makes differences more pronounced.

To assess prion curability by the Hsp104 inhibitor, guanidine hydrochloride (GuHCl) [35,66], yeast colonies were replica-plated on YPD plates containing 5 mM guanidine hydrochloride (GuHCl) for three consecutive passages (~20–40 generations total), followed by streaking out on YPD, colony purifying and analyzing individual colonies.

*Escherichia coli* cultures were grown in LB medium [67] (1% tryptone, 0.5% yeast extract, and 1% NaCl) at 37 °C. Ampicillin was added at final concentration of 100 µg/mL to select for plasmids.

All solid media contained 1.5% agar.

### 4.2. Plasmids

All plasmids used in this study are listed in Table S14. Most constructs were centromeric *S. cerevisiae–E. coli* shuttle vectors, typically based on empty vectors pRS315 (*LEU2*) and pRS316 (*URA3*), kindly provided by P. Hieter [68]. The plasmid pRS315-Pub1-mCherry was constructed by inserting the 2.6 kb *Hin*dIII-*Sac*I fragment containing the *PUB1-mCHERRY* gene under the endogenous *P_PUB1_* promoter from the plasmid pRP166, kindly provided by R. Parker [42], into pRS315 cut with the same enzymes. The plasmid pRS316-PCUP1-Sup35NM_SC_-CFP encoding the Sup35NM protein fused to cyan fluorescent protein (CFP) was a gift from S.P. Zadorsky. Plasmid pRS316-PCUP1-Sup35NSc-CFP, encoding the Sup35N protein fused to Cerulean, an improved brighter derivative of CFP, was constructed by inserting the *Not*I-*Sac*I *CFP* fragment, amplified from the plasmid p315-SFP1-Cerulean (provided by A. Matveenko [69]) using the primers listed in Table S15, into p316CUP1-Sup35NSc-YFP [16] cut with the same enzymes, thus replacing the *YFP*-coding sequence. All deletion derivatives of p316CUP1-Sup35NM-YFP and deletion derivatives ΔR1-3, ΔR4-6′ and ΔR1-6′ of p316CUP1-Sup35N-YFP were generated by PCR, amplifying an original plasmid with primers (Table S15) flanking the respective regions to be deleted and using a high-fidelity DNA polymerase (Accu Prime Pfx, Thermo Fisher Scientific, Waltham, MA, USA), or Q5, New England Biolabs, Inc. (Ipswich, MA, USA)). Following amplification, template plasmid DNA was eliminated by digesting with *Dpn*I, and amplified products were ligated using T4 DNA ligase (Thermo Fisher Scientific). Deletion derivatives *ΔNQ*, *ΔR4-6′CTN*, and *ΔCTN* of the plasmid p316CUP1-Sup35N-YFP were generated by PCR, amplifying respective *SUP35N* fragments with deletions from the p316CUP1-Sup35NM-YFP-based deletion plasmids with primers introducing *Bam*HI and *Sac*II restriction sites (Table S15) and inserting them into the p316CUP1-Sup35NM-YFP plasmid digested with the same enzymes, thus replacing the WT *SUP35NM* fragment to Sup35N with corresponding deletion. Notably, the centromeric and multicopy plasmids bearing *ΔNQ* retained the first two codons (M and S) to achieve translation initiation and higher protein stability; the latter assumption turned out to be incorrect, as explained in the Results; thus, integrative plasmids with *ΔNQ* retained only the initiator M codon.

Integrative plasmids bearing deletion derivatives of full-length *SUP35* (except *ΔNQ*, *ΔCTN* and *ΔR4-6′CTN*) under the control of the endogenous *P_SUP35_* promoter were generated on the basis of integrative plasmid pFL34-Sup35, a derivative of the *URA3* plasmid pFL34 [70], containing the *SUP35* gene under the control of the endogenous *P_SUP35_* promoter, and using the same PCR-based strategy as described above. The plasmid pFL34-Sup35 was generated by moving the 3.7 kb *Sph*I-*Xba*I *P_SUP35_-SUP35* fragment from plasmid pYCH-U2 [68] into pFL34. The integrative plasmid pFL34-Sup35ΔR4-6′CTN was generated by co-inserting two fragments into the plasmid pFL34, as described below. The first fragment comprising the *P_SUP35_* promoter and the *SUP35N* ORF down to (and including) codon 74 was PCR-amplified from the plasmid pYCH-U2 [71], using AccuPrime Pfx DNA polymerase and respective primers (Table S15), and digested with *Sph*I to generate a fragment with a 5′ *Sph*I-produced cohesive end. The second fragment, corresponding to the *SUP35* ORF starting from codon 124 down to the stop codon, was PCR-amplified from the same template plasmid using the same polymerase and a second set of phosphorylated primers and digested with *Xba*I to generate a 3′ *Xba*I-produced cohesive end. The plasmid pFL34 was then digested with *Sph*I and *Xba*I and ligated with both fragments, thus reconstructing the *SUP35*-coding sequence lacking codons 75–123. The ΔCTN deletion was initially generated in the plasmid pKS-PSUP35-SUP35 (a derivative of pBluescript II KS (+) [72] with the *SUP35* gene under its endogenous promoter constructed S.P. Zadorsky), using PCR with respective primers (Table S15) as described above. Then, the *Xho*I-*Xba*I fragment including the whole *sup35ΔCTN* cassette with a promoter was cut off and ligated into pFL34, digested with *Sal*I and *Xba*I.

To create multicopy plasmids encoding Sup35NMΔNQ-YFP and Sup35NΔNQ-YFP chimeric constructs placed under the *P_CUP1_* promoter, *Bam*HI-*Sac*I fragments respectively from p316-CUP1-Sup35NMΔNQ-YFP and p316-CUP1-Sup35NΔNQ-YFP plasmids were ligated into pFL44s-Aβ-GFP [73] digested with the same enzymes, thereby replacing the Aβ-GFP cassette. Multicopy plasmids carrying the ΔR1-6′ and ΔR4-6′CTN deletions in Sup35NM-YFP were generated by replacing the *BamH*I-*Sac*I Sup35NΔNQ-YFP fragment of pFL44s-PCUP1-Sup35NΔNQ-YFP with the corresponding fragments excised from the centromeric plasmids. The centromeric pYCH-Sup35ΔNQ plasmid bearing the *sup35ΔNQ* allele under the control of the endogenous *P_SUP35_* promoter was constructed by deleting the NQ-coding region (2–39 aas) in pYCH-U2, using PCR-mediated amplification with respective primers (Table S15) as described above. The centromeric plasmid pYCH-Sup35ΔNQ-LEU2 was constructed by replacing the *URA3* gene with *LEU2* from pFL36 [70] via *Bgl*II digestion and ligation. The integrative plasmid pFL34-Sup35ΔNQ was generated by moving the 3.6 kb *Sph*I-*Xba*I *P_SUP35_–sup35ΔNQ* fragment from pYCH-Sup35ΔNQ into pFL34. The centromeric pCUP1-Sup35ΔNQ plasmid, carrying the *sup35ΔNQ* allele under the control of the *P_CUP1_* promoter, was constructed by PCR, amplifying the whole derivative of the pmCUP1 plasmid bearing the *SUP35* ORF starting from position 41 (and excluding another insert replacing the NQ-coding region in that plasmid) and using 5′-phosphorylated primers (Table S15), followed by self-ligation to generate a plasmid carrying a deletion derivative of *SUP35* that lacks codons 2–40 and is placed under the *P_CUP1_* promoter. The *Xho*I-*Sac*I fragment of the plasmid was transferred into the pRS315 (Leu2) plasmid. All constructs have been confirmed by digestion and sequencing. All shuttle plasmids were propagated in standard *E. coli* strains XL1-Blue [74], XL10-Gold (Agilent Technologies, Inc. (Santa Clara, CA, USA)), or DH5α. Yeast cells were transformed according to the standard procedure [67].

### 4.3. Yeast Strains

The *Saccharomyces cerevisiae* strains used in this study are listed in Table S16. Strains GT12 [16] ([*PIN*^+^]) and AB190 ([*pin*^−^]) [16], carrying the allele *sup35ΔNM* with a deletion of the Sup35NM-coding region, are isogenic derivatives of 74-D694 [75]. All other strains are haploid isogenic derivatives of the GT81 series [76]. Initially, [*PIN*^+^ *psi*^−^] strains were constructed; the [*pin*^−^] derivative of the *sup35ΔM* strain was obtained by curing of the initially generated [*PIN*^+^ *psi*^−^] strain of the [*PIN*^+^] prion after growth on the medium with GuHCl. Chromosomal deletion alleles of *SUP35* were generated as previously described [77] using the respective *URA3* integrative plasmids described above, followed by pop-out in the result of homologous recombination selected on 5-FOA medium where *URA3*^+^ cells do not grow [34]. Integration of a plasmid into the *SUP35* locus was stimulated via linearizing this plasmid by *Mlu*I digestion before transformation. Replacements at the *SUP35* locus were verified by PCR and sequencing. Among *sup35N* deletion derivatives, *sup35ΔNQ* replacement was generated first, producing the Ade^+^ strain due to decreased levels of the Sup35ΔNQ protein (see above). All other replacements by *sup35N* deletion alleles were generated in the *sup35ΔNQ* strain so that the disappearance of the Ade^+^ phenotype helped to choose colonies for sequence analysis.

### 4.4. Analysis of Prion Induction and Mitotic Stability

To nucleate [*PSI*^+^] formation, individual yeast [*PIN*^+^ *pin*^−^] transformants with respective plasmids were patched on the synthetic medium selective for these plasmids (in most cases, -Ura) and velveteen replica-plated onto the same medium with the addition of 100 µM CuSO_4_ (except cases where prion formation was assessed at background levels of Cu^++^, as in Figure 6C), followed by replica plating onto –Ade medium with background levels of Cu^++^. Inheritance of the Ade^+^ phenotype in colonies grown on –Ade and presumably containing the [*PSI*^+^] prion was characterized further according to a well-established procedure (Figure S4A). Specifically, maintenance of the Ade^+^ phenotype after elimination of inducing *URA3* plasmids (coding for Sup35N/NM-YFP or Sup35NM-YFP, either WT or with respective deletions) via counterselection on 5-FOA was checked by velveteening Ade^+^ colonies onto 5-FOA medium. After 2 days of growth, colonies were colony-purified on YPD medium, individual colonies were patched on a YPD master plate, and after 2 days of growth, replica-plated onto –Ade. In parallel, colonies were replica-plated to identify spontaneously appearing petite (Pet^−^) derivatives losing mitochondrial DNA (due to their slow growth, they were excluded from further analysis) onto YPD medium and onto YPD with 5 µM GuHCl (followed by two subsequent replica platings onto fresh plates of the same medium, with 1–3 days of growth after each replica plating). After these passages, cultures grown on YPD and on YPD with 5 mM GuHCl were colony-purified on YPD and rechecked again for color on YPD and the retention of the Ade^+^ phenotype on –Ade. At least 8 subcolonies were tested for each original Ade^+^ colony in each condition. The stringency of [*PSI*^+^] strains in colonies retaining the GuHCl-curable prion was assessed by the intensity of growth on –Ade and lightness of color on YPD.

### 4.5. Fluorescent Microscopy

For FM analysis, yeast cultures were grown overnight with shaking at 200 rpm in liquid synthetic medium selective for respective plasmids, diluted to OD_600_ = 0.2 by fresh medium, and grown for another 24 h. Where indicated, CuSO_4_ (100 µM) was added to induce the *P_CUP1_* promoter. For hyperosmotic stress, cells were pelleted, resuspended in H_2_O with 1 M KCl, incubated for 10–20 min, and immediately used for imaging with a BX41 (Olympus Corporation, Tokyo, Japan) microscope with a 100×/NA 1.5 oil objective and an Olympus DP-71 color digital camera or a Leica DM6000B microscope (Leica Microsystems GmbH, Wetzlar, Germany) with a 100×/1.30 oil ph3 (UplanFl) objective.

### 4.6. Protein Analysis

For the analysis of protein levels, yeast cultures were grown for 24 h in YPD or synthetic medium (selective for plasmids), starting at OD_600_ = 0.2, and harvested from 1.5 mL of medium by centrifugation at 3000× *g* for 5 min. Proteins were extracted as described previously [78] with minor modifications. Cell pellets were pretreated with 2.0 M LiAc followed by 0.4 M NaOH for 5 min on ice, with centrifugation between each step before solution replacement, then resuspended in 100 µL SDS-PAGE sample buffer (60 mM Tris-HCl pH 6.8, 2% SDS, 10% glycerol, 2% β-mercaptoethanol and 0.002% bromophenol blue) and boiled for 5 min. Lysates were cleared by centrifugation and used for SDS-PAGE, with 8–12% (depending on protein size) separating gel and 4% stacking gels, in Tris-glycine-SDS running buffer (25 mM Tris, 192 mM glycine, 0.1% SDS, pH 8.3), followed by electrotransfer to PVDF or nitrocellulose membranes (0.45 µm; ThermoFisher Scientific). Membranes were blocked with either 5% nonfat milk or 2% Amersham ECL Prime Blocking Reagent (GE Healthcare, Chicago, IL, USA) and incubated with antibodies; proteins were visualized using Amersham ECL detection reagents (Cytiva, Marlborough, MA, USA). Depending on the protein, rabbit anti-GFP (Evrogen (Moscow, Russia), Cat# AB011, RRID:AB_2892560 or Sigma-Aldrich (Burlington, MA, USA), Cat# G1544, RRID:AB_439690) or chicken polyclonal anti-GFP (Abcam (Cambridge, UK), Cat# ab13970, RPID: AB_300798), rabbit polyclonal anti-Sup35C (gift from D. Bedwell), rat monoclonal anti-Sup35M (4A5; gift from I. Vorberg), or rabbit polyclonal anti-Sup35 (SE4290, [79]) primary antibodies were used. Secondary HRP-conjugated antibodies were goat anti-rabbit IgG (Sigma-Aldrich, Cat# A6154, RRID:AB_258284, or Abcam, Cat# ab97051, RPID: AB_10679369), HRP-linked donkey anti-rabbit IgG (Cytiva, Cat# NA934, RRID:AB_772206), and anti-rat IgG or goat anti-chicken IgY (H&L) (Abcam, Cat# ab6877).

To quantify protein levels, band intensities were measured using ImageJ (version 1.54p; NIH, Bethesda, MD, USA) or Image Lab (version 6.1.0; Bio-Rad Laboratories, Inc., Hercules, CA, USA) software. Signals were normalized to total protein loading, assessed either by TCE labeling (2,2,2-trichloroethanol, 1% *v*/*v*) incorporated into the polyacrylamide gel and visualized after transfer to nitrocellulose or PVDF membranes, or by Coomassie staining of the membrane. TCE fluorescence was activated by UV irradiation at 254 nm using an E-box VX5 (Vilber Lourmat SAS, Collégien, France) or GelDoc-It^TM^ 300 (UVP, LLC, Upland, CA, USA). For normalization, three TCE-positive bands per lane were quantified and averaged.

### 4.7. Statistical Analysis

In most experiments error bars represent standard deviations (SDs). Where specifically indicated, error bars represent standard errors of proportion (SEs), calculated as *SE* = √(*p*(*1* − *p*)/*n*), where *p* is the proportion of a given class and *n* is the total number of cells analyzed. Statistical significance was determined using Student’s *t*-test unless stated otherwise. Differences with *p* ≤ 0.05 were considered statistically significant. Significance levels are indicated as follows: * *p* ≤ 0.05, ** *p* ≤ 0.01, *** *p* ≤ 0.001. For data shown in Figure 4B,C, and respective Supplementary Tables S7 and S8, Firth penalized binomial logistic regression was applied. Paired experiments were always used for assessing the impact of osmotic stress by FM, with measurements of the same sample made before and after treatment within the same sample. As analyses were conducted in actively dividing cultures, this resulted in variability between independent biological replicates in some cases. However, the difference between positive and negative controls and comparisons between various derivatives were always maintained within each experiment, despite systematic overall variability between experiments. In such cases, a representative experiment is shown in a figure, and quantitative data from all replicates are provided in the Supporting Information Tables.

## 5. Conclusions

While the Sup35M domain of the intrinsically disordered region (IDR) of the *Saccharomyces cerevisiae* release factor Sup35 (eRF3) is dispensable for prion formation and prion propagation by the chaperone machinery, it modifies interactions with other proteins in an amyloid form. Various subregions of the Sup35N domain of the IDR exhibit partly overlapping or redundant but distinct impacts on its abilities to undergo osmotic-stress-inducible liquid–liquid phase separation (LLPS) in the absence of pre-existing prions, form amyloid fibrils in the presence of the Rnq1 prion, nucleate the [*PSI*^+^] prion, and propagate the heritable [*PSI*^+^] prion in cell generations. The region of oligopeptide repeats (OPR) plays a crucial role in LLPS, with deletion of its N-proximal portion having the strongest effect. The N-terminal NQ-rich stretch and then-proximal portion of the OPR are most important for amyloid formation and prion nucleation, while both the OPR and C-terminal region (CTN) are major players in chaperone-mediated prion propagation and inheritance. Contrary to some previous reports, the NQ stretch is not absolutely required for prion nucleation in yeast and has the least pronounced impact on prion propagation and heritability, which eliminates a seeming contradiction between aggregation behavior of the Sup35 IDR in yeast and the mammalian cell environment. Previous results were possibly influenced by the fact that elimination of the NQ stretch decreases protein levels, implicating this region in the control of proteolytic stability. Overall, this data supports the notion that liquid–liquid and liquid–solid transitions of the Sup35 IDR are controlled by specific (although partly redundant) sequence motifs, rather than solely by amino acid composition. Further studies are needed to decipher molecular mechanisms controlling these processes.

## Data Availability

All data supporting the findings of this study are available within the article and its Supplementary Materials. Original data, yeast strains, and plasmids generated in this study are available from the corresponding author upon reasonable request.

## Supplementary Materials

The following supporting information can be downloaded at https://www.mdpi.com/article/10.3390/ijms27177516/s1.
