# Supplementary material for: Deletion Analysis of Phase Separation, Amyloid Formation and Prion Propagation by the Intrinsically Disordered Region of Yeast Sup35 Protein
Source: Int J Mol Sci. 2026 Aug 22;27(17):7516. doi: 10.3390/ijms27177516 (PMC13566377; doi:10.3390/ijms27177516)
Supplement: Supplementary file 1 [file ijms-27-07516-s001.zip › 2026 Grizel et al Suppl Figs Final 07-21.pdf]

Deletion analysis of phase separation, amyloid formation and prion propagation by the intrinsically disordered region of yeast Sup35 protein

Anastasia V. Grizel, Natalia A. Gorsheneva, Ismat Jahan Anee, Kristupas Paulius, Konstantin Y. Kulichikhin, Aleksandr A. Rubel and Yury O. Chernoff

Supplementary Figures

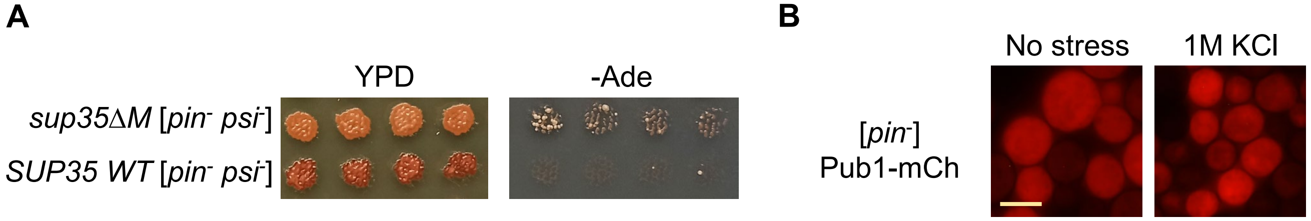

Figure S1. Additional data for the experiments with *sup35ΔM* strain and Pub1 aggregation. (A) Deletion of Sup35M (*sup35ΔM*) results in reduced accumulation of red pigment on YPD medium and background growth with intense papillation on medium lacking (–Ade) compared to the isogenic WT [*pin*$^-$ *psi*$^-$] strain. (B) Pub1-mCherry (Pub1-mCh) does not form detectable condensates in response to osmotic stress (1M KCl, 20 min) in [*pin*$^-$] *sup35ΔNM* cells which do not overexpress Sup35N/NM-based constructs. Scale bar corresponds to 5 μm.

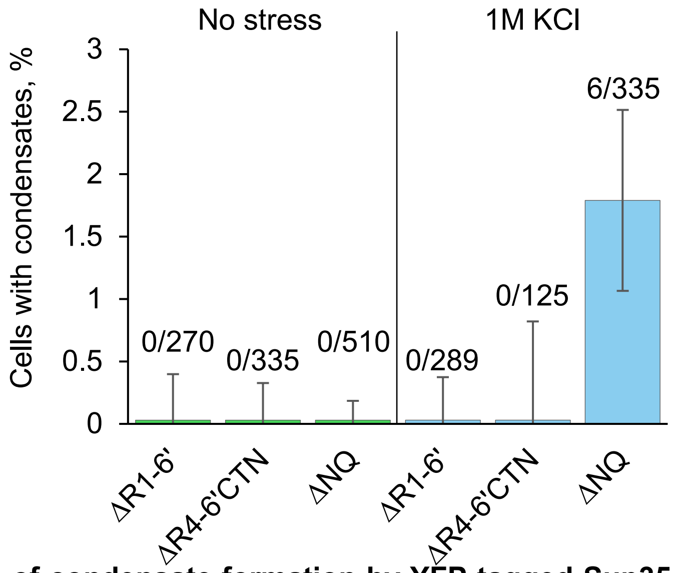

Figure S2. Comparison of condensate formation by YFP-tagged Sup35NM-based ΔR1–6′, ΔR4–6′CTN, and ΔQN derivatives expressed from multicopy plasmids in a [*pin*$^-$*psi*$^-$] strain. Percentage of cells with condensates are indicated on Y axis; numbers above the bars show numbers of condensate-containing cells (before /) out of total number of cells (after /). Error bars correspond to SEs.Cultures were grown in the presence of 100 μM $CuSO_4$ for 24 hrs. Osmotic stress was induced by 1M KCl in H_2O for 10 min.

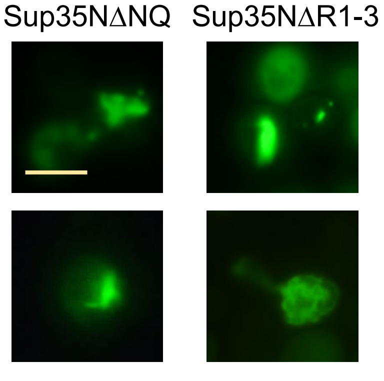

Figure S3. Abnormally shaped aggregates formed by ΔNQ and ΔR1–3 deletions. Images of cells expressing Sup35N-YFP with respective deletions and forming abnormal aggregates are shown after 24 hrs of overexpression in the presence of 100 μM $CuSO_4$. Scale bar corresponds to 10 μm.

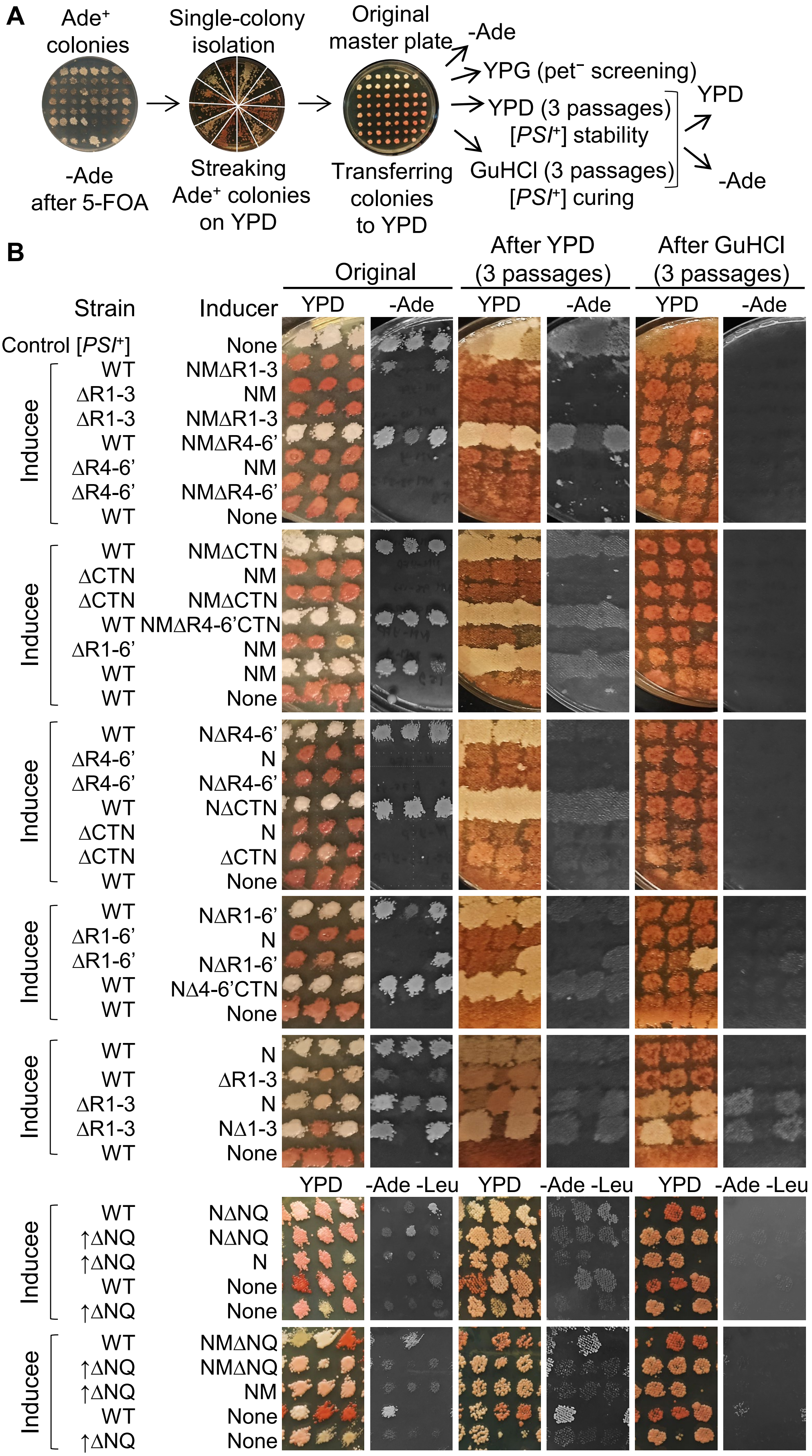

Figure S4. Analysis of Ade+ colonies induced in WT strain and strains with deletions within *SUP35N* domain. (A) Overview of the experimental workflow. (B) Analysis of Ade^{+} colonies after growth on 5-FOA medium according to the scheme shown in panel (A). Representative colonies of WT and deletion variants are shown, with the strong [*PSI*$^{+}$] control in the top row. Differences in [*PSI*$^{+}$] variants could be seen by color on YPD and by intensity of growth on -Ade. ↑ΔNQ – the strain bearing extra *sup35ΔNQ* allele on *LEU2* plasmid under the P_{CUP1} promoter.
