# Supplementary material for: Deletion Analysis of Phase Separation, Amyloid Formation and Prion Propagation by the Intrinsically Disordered Region of Yeast Sup35 Protein
Source: Int J Mol Sci. 2026 Aug 22;27(17):7516. doi: 10.3390/ijms27177516 (PMC13566377; doi:10.3390/ijms27177516)
Supplement: Supplementary file 1 [file ijms-27-07516-s001.zip › 2026 Grizel et al Suppl Tables Revised 08-15.pdf]

Deletion analysis of phase separation, amyloid formation and prion propagation by the intrinsically disordered region of yeast Sup35 protein

Anastasia V. Grizel, Natalia A. Gorsheneva, Ismat Jahan Anee, Kristupas Paulius, Konstantin Y. Kulichikhin, Aleksandr A. Rubel and Yury O. Chernoff

Supplementary Tables

Table S1. Comparison of Sup35 protein levels in [*PIN*+ *psi*-] WT and *sup35ΔM* strains. (Data for Figure 2, B and C.)

Strain	Replicate	Normalized intensity	Mean ± SD
WT	1	1.3	1.00 ± 0.2
	2	0.91	
	3	0.88	
	4	0.91	
sup35ΔM	1	0.8	0.71 ± 0.08
	2	0.62	
	3	0.75	
	4	0.67	

Data are normalized relative to mean WT Sup35 levels.

Table S2. Levels of Sup35 protein in [*PIN*+ *psi*-] strains bearing deletions in the chromosomal copy of *SUP35*. (Data for Figure 3B.)

Strain/allele	Replicate 1	Replicate 2	Replicate 3	Mean ± SD
WT	1.00	1.00	1.00	1.00
sup35ΔR1-3	0.90	1.98	0.79	1.22 ± 0.66
sup35ΔR4-6′	1.27	0.87	0.77	0.97 ± 0.27
sup35ΔR1-6′	1.03	1.06	0.35	0.81 ± 0.40
sup35ΔR4-6′CTN	1.43	1.25	0.80	1.16 ± 0.32
sup35ΔCTN	1.14	0.63	1.32	1.03 ± 0.36

Data are normalized relative to WT Sup35 levels in each replicate.

Table S3. Comparison of Sup35 protein levels in [*pin*- *psi*-] WT and *sup35ΔNQ* strains. (Data for Figure 3D.)

Strain	Replicate	Normalized intensity	Mean ± SD
WT	1	1.06	1.00 ± 0.05
	2	0.96	
	3	0.98	
sup35ΔNQ	1	0.71	0.50 ± 0.19
	2	0.42	
	3	0.35	

Data are normalized relative to mean WT Sup35 levels.

Table S4. Protein levels of Sup35N–YFP and Sup35NM-YFP derivatives with deletions in the Sup35N produced in the [*pin⁻ psi⁻*] *sup35ΔNM* strain in the presence of 100 μM $CuSO_4$. (Data for Figure 3F.)

Protein	Number of replicates	Range	Mean ± SD
Sup35N-YFP	13	0.63–1.43	1.00 ± 0.25
Sup35NΔNQ-YFP	9	0.01–0.32	0.12 ± 0.11
Sup35NΔR1-3-YFP	5	0.32–1.27	0.90 ± 0.37
Sup35NΔR1-6'-YFP	6	1.03–2.16	1.35 ± 0.43
Sup35NΔR4-6'CTN-YFP	5	0.47–2.46	1.13 ± 0.78
Sup35NΔR4-6'-YFP	6	1.14–2.18	1.47 ± 0.37
Sup35NΔCTN-YFP	5	0.63–1.24	0.98 ± 0.24
Sup35NM-YFP	19	0.59–1.70	1.00 ± 0.24
Sup35NMΔNQ-YFP (CEN)	15	0.00–0.38	0.14 ± 0.13
Sup35NMΔR1-3-YFP	5	0.55–1.14	0.89 ± 0.27
Sup35NMΔR1-6'-YFP	6	0.44–1.82	1.11 ± 0.59
Sup35NMΔR4-6'CTN-YFP	5	0.36–2.10	0.98 ± 0.71
Sup35NMΔR4-6'-YFP	6	0.33–1.81	1.00 ± 0.50
Sup35NMΔCTN-YFP	5	0.15–1.51	0.88 ± 0.51

Data are normalized relative to means of respective WT controls (Sup35NM-WT for Sup35NM derivatives, and Sup35N-WT for Sup35N derivatives).

Table S5. Levels of Sup35ΔNQ protein encoded by the respective chromosomal allele in the absence and presence of the *sup35ΔNQ* plasmids, as compared to WT control. (Data for Figure 3G.)

Strain	Plasmid	Technical replicate	Normalized value	Mean ± SD
WT	pRS315	1	1.00	1.00
		2	1.00	
		3	1.00	
sup35ΔNQ	pRS315	1	0.36	0.34 ± 0.02
		2	0.32	
		3	0.36	
sup35ΔNQ	pYCH-Sup35ΔNQ-LEU2	1	0.47	0.48 ± 0.02
		2	0.50	
		3	0.48	
sup35ΔNQ	pCUP1-Sup35ΔNQ	1	1.07	1.11 ± 0.05
		2	1.17	
		3	1.11	

Data are normalized relative to WT Sup35 levels in each replicate.

Table S6. Levels of ΔNQ-YFP proteins expressed from multicopy (2μ) and centromeric (CEN) plasmids in in the [*pin*⁻ *psi*⁻] *sup35ΔNM* strain. (Data for Figure 3I.)

**Protein (plasmid type)**	**Replicate**	**Normalized intensity**	**Mean ± SD**
Sup35NΔNQ-YFP (CEN)	1	0.13	0.20 ± 0.12
	2	0.06	
	3	0.28	
	4	0.32	
Sup35NΔNQ-YFP (2μ)	1	2.07	1.87 ± 0.81
	2	1.01	
	3	1.49	
	4	2.90	
Sup35N(WT)-YFP (CEN)	1	1.09	1.00 ± 0.33
	2	0.75	
	3	0.73	
	4	1.43	
Sup35NMΔNQ-YFP (CEN)	1	0.00	0.15 ± 0.13
	2	0.10	
	3	0.28	
	4	0.23	
Sup35NMΔNQ-YFP (2μ)	1	2.67	2.57 ± 0.97
	2	1.43	
	3	2.38	
	4	3.79	
Sup35NM(WT)-YFP (CEN)	1	1.06	1.00 ± 0.08
	2	1.03	
	3	0.88	
	4	1.04	

Data are normalized relative to means of respective WT controls (Sup35NM-WT for Sup35NM derivatives, and Sup35N-WT for Sup35N derivatives).

Table S7. Formation of Sup35N-YFP condensates in [*pin⁻ psi⁻*] *sup35ΔNM* cells before and after 1M KCl treatment. (Data for Figure 4B.)

Trial	Protein deletion	No stress		1M KCl	
		Cells with puncta	Total	Cells with puncta	Total
1	WT	448 (37.12%)	1207	521 (47.11%)	1106
	ΔR1-3	7 (0.33%)	2108	20 (1.93%)	1038
	ΔR4-6'	25 (1.57%)	1595	55 (6.29%)	875
	ΔR1-6'	0	1976	2 (0.10%)	1972
	ΔR4-6'CTN	0	1415	12 (0.77%)	1560
	ΔCTN	79 (5.15%)	1533	97 (11.60%)	836
2	WT	269 (48.73%)	552	512 (69.28%)	739
	ΔR1-3	1 (0.11%)	939	32 (4.02%)	797
	ΔR4-6'	57 (6.51%)	875	124 (13.33%)	930
	ΔR4-6'	2 (0.11%)	1748	22 (2.74%)	802
	ΔR4-6'	0	1017	26 (2.80%)	927
	ΔR1-6'	0	516	0	1404
	ΔR4-6'CTN	0 (0%)	983	16 (1.37%)	1165
	ΔCTN	33 (5.86%)	563	171 (15.05%)	1136
3	WT	18 (2.81%)	640	216 (26.31%)	821
	ΔR1-3	0	1036	10 (0.89%)	1125
	ΔR4-6'	4 (0.23%)	1705	35 (2.68%)	1306
	ΔR1-6'	0	776	0	1332
4	WT	39 (5.90%)	661	240 (19.56%)	1227
	ΔR1-3	0	1014	6 (0.76%)	791
	ΔR4-6'	5 (0.45%)	1112	13 (1.74%)	745
	ΔR1-6'	0	1063	0	1216
5	WT	205 (34.75%)	590	515 (47.86%)	1076
	ΔNQ(2μ)	19 (5.44%)	349	41 (13.36%)	307
	ΔNQ(2μ)	29 (10.25%)	283	44 (17.46%)	252
	ΔR4-6'CTN	0	558	5 (0.42%)	1199
	ΔR1-6'	0	705	0	1759
6	WT	133 (15.15%)	878	107 (26.35%)	406
	ΔCTN	28 (4.71%)	594	104 (13.77%)	755
	ΔR4-6'CTN	0	531	0	646
7	WT	85 (14.05%)	605	127 (25.40%)	500
	ΔR1-3	0	514	6 (1.85%)	325
8	WT	222 (42.44%)	523	422 (54.80%)	770
	ΔNQ(2μ)	5 (2.96%)	169	10 (5.62%)	178
Total	WT	1419 (25.09%)	5656	2660 (40.03%)	6645
	ΔNQ(2μ)	53 (6.62%)	801	95 (12.89%)	737
	ΔR1-3	8 (0.14%)	5611	74 (1.82%)	4076
	ΔR4-6'	93 (1.15%)	8052	275 (4.92%)	5585
	ΔR1-6'	0	5036	2 (0.03%)	7683
	ΔR4-6'CTN	0	3487	33 (0.72%)	4570
	ΔCTN	140 (5.20%)	2690	372 (13.64%)	2727

Trials 1 and 5 were used for diagram on Figure 4B.

Table S8. Formation of Sup35NM-YFP condensates in [*pin⁻ psi⁻*] *sup35ΔNM* cells before and after 1M KCl treatment continuation. (Data for Figure 4C.)

Trial	Protein	No stress		1M KCl	
		Cells with puncta	Total	Cells with puncta	Total
1	WT	39 (5.34%)	730	251 (28.85%)	870
	ΔNQ(2μ)	0	510	12 (3.50%)	343
	ΔR1-3	0	709	0	967
	ΔR4-6'	0	1124	19 (2.08%)	914
	ΔR1-6'	0	1028	0	902
	ΔR4-6'CTN	0	1069	0 (0.00%)	594
	ΔCTN	0	1019	61 (5.54%)	1101
2	WT	133 (10.35%)	1285	553 (44.24%)	1250
	ΔR4-6'	0	622	38 (5.61%)	677
	ΔR1-6'	0	913	0	973
	ΔR4-6'CTN	0	581	0	955
	ΔCTN	0	795	98 (10.29%)	952
3	WT	3 (0.74%)	408	50 (4.45%)	1123
	WT	4 (0.66%)	602	67 (5.00%)	1339
	ΔR1-3	0	950	0	1153
	ΔR1-3	0	901	2 (0.11%)	1775
	ΔR4-6'	0	885	6 (0.51%)	1167
	ΔR4-6'	2 (0.17%)	1210	2 (0.10%)	1959
	ΔR1-6'	0	856	2 (0.07%)	2833
	ΔR1-6'	0	713	0	1298
4	WT	3 (0.23%)	1303	91 (6.70%)	1359
	ΔR1-3	0	1049	0	1496
	ΔR4-6'	0	734	9 (0.30%)	2998
	ΔR1-6'	0	1176	0	720
5	WT	6 (0.47%)	1274	39 (5.51%)	708
	ΔR1-3	0	737	0	768
	ΔR4-6'	0	869	12 (1.17%)	1024
	ΔR1-6'	0	1073	0	1643
6	WT	5 (1.58%)	317	20 (3.91%)	512
	ΔR4-6'CTN	0	178	0	530
	ΔR4-6'CTN	0	457	0	464
	ΔCTN	0	269	4 (0.76%)	523
	ΔCTN	0	240	5 (0.87%)	574
7	WT	6 (0.58%)	1028	92 (10.13%)	908
	ΔR4-6'CTN	0	1321	0	1405
	ΔR4-6'CTN	0	1500	0	1888
	ΔCTN	0	1691	32 (1.79%)	1789
	ΔCTN	0	1684	39 (2.37%)	1645

Trial 1 is used for the diagram on Figure 4C.

Continuation is on the next page.

Table S8. Formation of Sup35NM-YFP condensates in [*pin⁻ psi⁻*] *sup35ΔNM* cells before and after 1M KCl treatment. (Continuation.)

Trial	Protein	No stress		1M KCl	
		Cells with puncta	Total	Cells with puncta	Total
8	WT	18 (2.76%)	651	52 (10.51%)	495
	WT	7 (0.43%)	1621	146 (6.84%)	2134
	ΔR4-6'CTN	0	1101	0	1267
	ΔR4-6'CTN	0	1249	0	1392
	ΔCTN	0	1146	26 (1.89%)	1374
	ΔCTN	0	926	65 (2.60%)	2498
9	WT	6 (0.58%)	1028	92 (10.13%)	908
	ΔNQ(2μ)	0	607	4 (1.74%)	230
	ΔNQ(2μ)	0	545	4 (1.82%)	220
	ΔNQ(2μ)	0	532	2 (0.90%)	223
	ΔNQ(2μ)	0	136	0	107
Total	WT	230 (2.24%)	10247	1453 (12.52%)	11606
	ΔNQ(2μ)	0	2330	22 (1.96%)	1123
	ΔR1-3	0	4346	2 (0.03%)	6159
	ΔR4-6'	2 (0.04%)	5444	86 (0.98%)	8739
	ΔR1-6'	0	5759	2 (0.02%)	8369
	ΔR4-6'CTN	0	7456	0	8493
	ΔCTN	0	7770	330 (3.16%)	10456

Table S9. Formation of assemblies by YFP-tagged Sup35N and NM derivatives in [*PIN*$^{+}$ *psi*$^{-}$] yeast strain, grown for 24 hrs with addition of 100 μM $CuSO_4$. (Data for Figure 5, A and B.)

Protein derivative	Replicates	Dots		Filaments		Total (dots + filaments)	
		Range	Mean ± SD	Range	Mean ± SD	Mean ± SD	Total cells scored
Sup35N	5	0.35–4.90%	2.62 ± 2.07%	43.03–67.57%	55.10 ±10.62%	57.72 ± 9.21%	3151
Sup35NΔNQ (2μ)	4	6.07–10.42%	8.16 ± 2.20%	2.78–4.17%	3.31 ± 0.65%	11.46 ± 2.82%	704
Sup35NΔR1-3	3	0.07–0.68%	0.31 ± 0.33%	0.23–1.70%	0.73 ± 0.84%	1.04 ± 0.67%	4807
Sup35NΔR4-6'	3	0.13–3.08%	2.05 ± 1.66%	16.71–18.30%	17.65 ± 0.83%	19.69 ± 1.66%	2948
Sup35NΔR1-6'	4	0.00–2.24%	1.07 ± 1.04%	0.00–0.98%	0.29 ± 0.46%	1.36 ± 1.28%	5034
Sup35NΔR4-6'CTN	4	0.00–2.44%	0.86 ± 1.09%	0.00–1.60%	0.67 ± 0.68%	1.53 ± 1.74%	4481
Sup35NΔCTN	4	0.71–3.47%	2.11 ± 1.57%	3.61–12.12%	8.08 ± 4.57%	10.18 ± 4.74%	3771
Sup35NM	5	2.93–6.25%	4.56 ± 1.50%	4.40–13.78%	10.82 ± 3.84%	15.37 ± 4.82%	2939
Sup35NMΔNQ (2μ)	5	0	0	0.00–3.28%	1.04 ± 1.50%	1.04 ± 1.50%	824
Sup35NMΔR1-3	4	0.00–0.17%	0.08 ± 0.09%	0.00–0.10%	0.03 ± 0.05%	0.11 ± 0.08%	4173
Sup35NMΔR4-6'	3	0.04–0.62%	0.27 ± 0.31%	0.27–2.99%	1.22 ± 1.53%	1.49 ± 1.84%	4227
Sup35NMΔR1-6'	4	0.00–0.00%	0.00 ± 0.00%	0.00–0.00%	0.00 ± 0.00%	0.00 ± 0.00%	3083
Sup35NMΔR4-6'CTN	6	0.00–2.69%	0.46 ± 1.09%	0.00–2.69%	0.59 ± 1.06%	1.06 ± 2.14%	6453
Sup35NMΔCTN	4	0.37–3.05%	1.15 ± 1.27%	1.59–4.94%	2.91 ± 1.61%	4.06 ± 2.30%	3535

Table S10. Analysis of Ade$^+$ colonies induced by various YFP-tagged Sup35N and NM derivatives in WT [*PIN*$^+$ *psi*$^-$] strain. (Data for Figure 7C.)

Inducer	Becoming Ade$^-$	Retaining Ade$^+$		Total
		Incurable	GuHCl-curable ([*PSI*$^+$])	
N-WT	2	0	12	14
NM-WT	0	0	14	14
Total	2 (7%)	0	26 (93%)	28
NΔR1-3	2	0	6	8
NMΔR1-3	14	0	9	23
Total	16 (52%)	0	15 (48%)	31
NΔR1-6′	0	1 (14%)	6 (86%)	7
NΔR4-6′CTN	0	0	8	8
NMΔR4-6′CTN	0	0	8	8
Total	0	0	16 (100%)	16
NΔR4-6′	0	0	8	8
NMΔR4-6′	0	0	8	8
Total	0	0	16 (100%)	16
NΔR4-6′	0	0	8	8
NMΔR4-6′	0)	0	7	7
Total	0	0	15 (100%)	15
NΔNQ (2μ)	1	0	11	12
NMΔNQ (2μ)	2	1	9	12
Total	3 (13%)	1 (4%)	20 (83%)	24

Table S11. Spectra of prion variants induced by various YFP-tagged Sup35N and NM derivatives in WT [*PIN*$^+$ *psi*$^-$] strain. (Data for Figure 7C.)

Inducer	Weak [*PSI*$^+$]	Intermediate [*PSI*$^+$]	Strong [*PSI*$^+$]	Total
N-WT	0	0	12	12
NM-WT	1	6	7	14
Total	1 (4%)	6 (23%)	19 (73%)	26
NΔR1-3	3	1	2	6
NMΔR1-3	6	0	3	9
Total	9 (60%)	1 (7%)	5 (33%)	15
NΔR1-6′	2 (33%)	0 (0%)	4 (67%)	6
NΔR4-6′CTN	0	0	8	8
NMΔR4-6′CTN	2	1	5	8
Total	2 (13%)	1 (6%)	13 (81%)	16
NΔR4-6′	2	0	6	8
NMΔR4-6′	0	1	7	8
Total	2 (13%)	1 (6%)	13 (81%)	16
NΔCTN	0	0	8	8
NMΔCTN	3	0	5	8
Total	3 (19%)	0	13 (81%)	16
NΔNQ (2μ)	7	3	1	11
NMΔNQ (2μ)	1	0	8	9
Total	8 (40%)	3 (15%)	9 (45%)	20

Table S12. Analysis of Ade$^+$ colonies induced by various YFP-tagged Sup35N and NM derivatives in [*PIN*$^+$ *psi*$^-$] strains with *sup35* deletion alleles in the chromosome. (Data for Figure 7C.)

Inducee	Inducer type	Inducer							
		Δ				WT			
		Become Ade$^-$	Remaining Ade$^+$		Total	Become Ade$^-$	Remaining Ade$^+$		Total
			Incurable	Curable ([*PSI*$^+$])			Incurable	Curable ([*PSI*$^+$])	
ΔR1-3	N	13	7	1	21	2	4	0	6
	NM	24	0	0	24	24	0	0	24
	Total	37 (82%)	7 (16%)	1 (2%)	45	26 (87%)	4 (13%)	0	30
ΔR4-6′	N	24	0	0	24	18	3	2	23
	NM	23	0	1	24	24	0	0	24
	Total	47 (98%)	0	1(2%)	48	42 (89%)	3 (6%)	2 (4%)	47
ΔR1-6′	N	7	1	0	8	24	0	0	24
	NM	14	0	0	14	18	0	0	18
	Total	21 (96%)	1 (4%)	0	22	42 (100%)	0	0	42
ΔCTN	N	23	0	1	24	22	1	0	23
	NM	23	0	1	24	22	0	1	23
	Total	46 (96%)	0	2 (4%)	48	44 (96%)	1 (2%)	1 (2%)	46
ΔNQ(2μ)	N	4	2	1	7	5	6	2	13
	NM	4	3	1	8	12	1	3	16
	Total	8 (53%)	5 (33%)	2 (13%)	15	17 (59%)	7 (24%)	5 (17%)	29

Table S13. Spectra of prion variants induced by various YFP-tagged Sup35N and NM derivatives in [*PIN*$^+$ *psi*$^-$] strains with *sup35* deletion alleles in the chromosome. (Data for Figure 7C.)

Inducee	Inducer type	Inducer composition							
		Δ				WT			
		Weak	Intermediate	Strong	Total	Weak	Intermediate	Strong	Total
ΔR1-3	N	1	0	0	1	NA	NA	NA	0
	Total	1	0	0	1	NA	NA	NA	0
ΔR4-6′	N	NA	NA	NA	0	2	0	0	2
	NM	1	0	0	1	NA	NA	NA	0
	Total	1	0	0	1	2	0	0	2
ΔCTN	N	1	0	0	1	NA	NA	NA	0
	NM	1	0	0	1	1	0	0	1
	Total	2	0	0	2	1	0	0	1
ΔNQ(2μ)	N	1	0	0	1	0	2	0	2
	NM	0	1	0	1	3	0	0	3
	Total	1	1	0	2	3	2	0	5

Table S14. Plasmids used in this study.

Plasmid name	Plasmid type	Yeast marker	Promoter	Expression cassette	Origin
pRS316	*CEN*	*URA3*	-	-	[68]
pRS315	*CEN*	*LEU2*	-	-	[68]
pRS316-PCUP1-Sup35NM$_{Sc}$-CFP	*CEN*	*URA3*	P_{CUP1}	*SUP35NM$_{SC}$-CFP*	This study
pRS316-PCUP1-Sup35N$_{Sc}$-CFP(Cerulean)	*CEN*	*URA3*	P_{CUP1}	*SUP35N$_{SC}$-Cerulean*	This study
pPub1-mCherry	*CEN*	*LEU2*	P_{PUB1}	*PUB1-mCHerry*	This study
p316CUP1-Sup35NM$_{Sc}$-YFP	*CEN*	*URA3*	P_{CUP1}	*SUP35NM-YFP*	[16]
p316CUP1-Sup35N$_{Sc}$-YFP	*CEN*	*URA3*	P_{CUP1}	*SUP35N-YFP*	[16]
p316CUP1-Sup35NMΔNQ-YFP	*CEN*	*URA3*	P_{CUP1}	*sup35NMΔNQ-YFP*	This study
p316CUP1-Sup35NΔNQ-YFP	*CEN*	*URA3*	P_{CUP1}	*sup35NΔNQ-YFP*	This study
p316CUP1-Sup35NMΔR1-3-YFP	*CEN*	*URA3*	P_{CUP1}	*sup35NMΔR1-3-YFP*	This study
p316CUP1-Sup35NΔR1-3-YFP	*CEN*	*URA3*	P_{CUP1}	*sup35NΔR1-3-YFP*	This study
p316CUP1-Sup35NMΔR4-6′-YFP	*CEN*	*URA3*	P_{CUP1}	*sup35NMΔR4-6′-YFP*	This study
p316CUP1-Sup35NΔR4-6′-YFP	*CEN*	*URA3*	P_{CUP1}	*sup35NΔR4-6′-YFP*	This study
p316CUP1-Sup35NMΔR1-6′-YFP	*CEN*	*URA3*	P_{CUP1}	*sup35NMΔR1-6′-YFP*	This study
p316CUP1-Sup35NΔR1-6′-YFP	*CEN*	*URA3*	P_{CUP1}	*sup35NΔR1-6′-YFP*	This study
p316CUP1-Sup35NMΔR4-6′CTN-YFP	*CEN*	*URA3*	P_{CUP1}	*sup35NMΔR4-6′CTN-YFP*	This study
p316CUP1-Sup35NΔR4-6′CTN-YFP	*CEN*	*URA3*	P_{CUP1}	*sup35NΔR4-6′CTN-YFP*	This study
p316CUP1-Sup35NMΔCTN-YFP	*CEN*	*URA3*	P_{CUP1}	*sup35NMΔCTN-YFP*	This study
p316CUP1-Sup35NΔCTN-YFP	*CEN*	*URA3*	P_{CUP1}	*sup35NΔCTN-YFP*	This study
pFL44s-CUP1-Sup35NMΔNQ-YFP	*2μ*	*URA3*	P_{CUP1}	*sup35NMΔNQ-YFP*	This study
pFL44s-PCUP1-Sup35NΔNQ-YFP	*2μ*	*URA3*	P_{CUP1}	*sup35NΔNQ-YFP*	This study
pFL44s-CUP1-Sup35NMΔR1-6′-YFP	*2μ*	*URA3*	P_{CUP1}	*sup35NMΔR1-6-YFP*	This study
pFL44s-CUP1-Sup35NMΔR4-6′CTN-YFP	*2μ*	*URA3*	P_{CUP1}	*sup35NMΔR4-6′CTN-YFP*	This study
pYCH-Sup35ΔNQ	*CEN*	*URA3*	P_{SUP35}	*sup35ΔNQ*	This study
pYCH-Sup35ΔNQ-LEU2	*CEN*	*LEU2*	P_{SUP35}	*sup35ΔNQ*	This study
pCUP1-Sup35ΔNQ	*CEN*	*LEU2*	P_{CUP1}	*sup35ΔNQ*	This study
pFL34-Sup35ΔNQ	*INT*	*URA3*	P_{SUP35}	*sup35ΔNQ*	This study
pFL34-Sup35ΔR1-3	*INT*	*URA3*	P_{SUP35}	*sup35ΔR1-3*	This study
pFL34-Sup35ΔR4-6′	*INT*	*URA3*	P_{SUP35}	*sup35ΔR4-6′*	This study
pFL34-Sup35ΔR1-6′	*INT*	*URA3*	P_{SUP35}	*sup35ΔR1-6′*	This study
pFL34-Sup35ΔR4-6′CTN	*INT*	*URA3*	P_{SUP35}	*sup35ΔR4-6′CTN*	This study
pFL34-Sup35ΔCTN	*INT*	*URA3*	P_{SUP35}	*sup35ΔCTN*	This study
pFL34-Sup35ΔM	*INT*	*URA3*	P_{SUP35}	*sup35ΔM*	This study
pKS-PSUP35-SUP35ΔCTN	*None*	*None*	P_{SUP35}	*sup35ΔCTN*	This study

Table S15. Oligonucleotide primers used in this study.

Name	Sequence 5′-3′	Used in construction of
F-Cerulean-NotI	GTTAGCGGCCGCTATGGTGAGCAAGGGCGAG	pRS316-PCUP1-Sup35N$_{SC}$-CFP
R-Cerulean-SacI	GTCTGAGCTCTTACTTGTACAGCTCGTCCATGCC	
pCup1_REV	PO_4-CATGGATCCTGATTGATTGATTG	p316CUP1-Sup35NMΔNQ-YFP
Sup35N_40F	PO_4-CAACCTGCAGGTGGGTACTACC	
F-Sup35_d75	PO_4-CCTCAAGGAGGCTATCAAC	p316CUP1-Sup35NMΔR1-3-YFP
R-Sup35_d39	PO_4 -TTGGGCTTGAGCATTGTAAG	p316CUP1-Sup35NΔR1-3-YFP
		pFL34-Sup35ΔR1-3
F-Sup35_d97	PO_4-CGTGGAAATTACAAAAACTTC	p316CUP1-Sup35NMΔR4-6′-YFP
R-Sup35N_d74	PO_4-ATTATACTGTTGCTGGTAACCGG	p316CUP1-Sup35NMΔR4-6′-YFP
		pFL34-Sup35ΔR4-6′
F-Sup35_d97	PO_4-CGTGGAAATTACAAAAACTTC	p316CUP1-Sup35NMΔR1-6′-YFP
		p316CUP1-Sup35NΔR1-6′-YFP
R-Sup35_d39	PO_4 -TTGGGCTTGAGCATTGTAAG	pFL34-Sup35ΔR1-6′
F-Sup35N_d98-123	PO_4-ATGTCTTTGAACGACTTTCAA AAGC	p316CUP1-Sup35NMΔR4-6′CTN-YFP
R-Sup35N_d74	PO_4-ATTATACTGTTGCTGGTAACCGG	pKS-PSUP35-SUP35ΔCTN
F-Sup35N_d98-123	PO_4-ATGTCTTTGAACGACTTTCAAAAGC	p316CUP1-Sup35NMΔCTN-YFP
R-Sup35N_d98-123	PO_4-GCCACCTTGTGGATTGAATTGC	
F-Sup35N-BamHI	ATCAGGATCCACAATGTCGGATTC	p316CUP1-Sup35NΔR4-6′CTN-YFP
R-Sup35N-d75-SacII	ATGCCCGCGGATTATACTGTTGCTGGTAACC	
F-Sup35N-d3-39	GAACGGATCCAAAATGTCCCAACCTGCAGGTGGG	p316CUP1-Sup35NΔNQ-YFP
R-Sup35N-SacII	TTCACCGCGGACCTTGAGACTGTGGTTGG	
F-Sup35N-BamHI	ATCAGGATCCACAATGTCGGATTC	p316CUP1-Sup35NΔCTN-YFP
R-Sup35N-d98-SacII	GCTACCGCGGGCCACCTTGTGGATTG	
pFL38R	ACACAGGAAACAGCTATG	pFL34-Sup35ΔR4-6′CTN
R-Sup35N_d74	PO_4-ATTATACTGTTGCTGGTAACCGG	
pFL38F	TAAGTTGGGTAACGCCAG	
F-Sup35N_d98-123	PO_4-ATGTCTTTGAACGACTTTCAAAAGC	
F-Sup35M	PO_4-TTTGGTGGTAAAGATCAC	pFL34-Sup35ΔM
R-Sup35M	PO_4-CATACCTTGAGACTGTGG	
Sup35N_PYCH-U2-1R	PO_4 -CATTGTTGCTAGTGG GCAGATATAGATG	pYCH-Sup35ΔNQ
Sup35N_40F	PO_4-CAACCTGCAGGTGGGTACTACC	
F-Sup35NRMC	PO_4-CCTGCAGGTGGGTACTACC	pCUP1-Sup35ΔNQ
pCUP1-REV	PO_4-CATGGATCCTGATTGATTGATTG	

Table S16. Yeast strains used in the study.

Strain name	Prion composition	Strain origin	Genotype
GT409	[*psi⁻ pin⁻*]	GT81	*MAT***a** *ade1-14 his3 leu2 lys2 trp1 ura3*
GT159	[*psi⁻ PIN⁺*]	GT81	*MAT***a** *ade1-14 his3 leu2 lys2 trp1 ura3*
GT81-1C	[*PSI⁺ PIN⁺*]	GT81	*MAT***a** *ade1-14 his3 leu2 lys2 trp1 ura3*
AB190	[*psi⁻ pin⁻*]	74-D694	*MAT***a** *ade1-14 his3 leu2 trp1 ura3 sup35ΔNM*
GT12	[*psi⁻ PIN⁺*]	74-D694	*MAT***a** *ade1-14 his3 leu2 trp1 ura3 sup35ΔNM*
AB233	[*psi⁻ pin⁻*]	GT81	*MAT***a** *ade1-14 his3 leu2 lys2 trp1 ura3 sup35Δ39*
AB235	[*psi⁻ PIN⁺*]	GT81	*MAT***a** *ade1-14 his3 leu2 lys2 trp1 ura3 sup35Δ39*
AB267	[*psi⁻ PIN⁺*]	GT81	*MAT***a** *ade1-14 his3 leu2 lys2 trp1 ura3 sup35Δ40-74*
AB269	[*psi⁻ PIN⁺*]	GT81	*MAT***a** *ade1-14 his3 leu2 lys2 trp1 ura3 sup35Δ75-97*
AB266	[*psi⁻ PIN⁺*]	GT81	*MAT***a** *ade1-14 his3 leu2 lys2 trp1 ura3 sup35Δ98-123*
AB236	[*psi⁻ PIN⁺*]	GT81	*MAT***a** *ade1-14 his3 leu2 lys2 trp1 ura3 sup35Δ75-123*
AB268	[*psi⁻ PIN⁺*]	GT81	*MAT***a** *ade1-14 his3 leu2 lys2 trp1 ura3 sup35Δ40-97*
AB270	[*psi⁻ PIN⁺*]	GT81	*MAT***a** *ade1-14 his3 leu2 lys2 trp1 ura3 sup35ΔM*
GT2489	[*psi⁻ pin⁻*]	GT81	*MAT***a** *ade1-14 his3 leu2 lys2 trp1 ura3 sup35ΔM*
